# Financial fluctuations anchored to economic fundamentals: A mesoscopic network approach

**DOI:** 10.1038/s41598-017-07758-9

**Published:** 2017-08-14

**Authors:** Kiran Sharma, Balagopal Gopalakrishnan, Anindya S. Chakrabarti, Anirban Chakraborti

**Affiliations:** 1 School of Computational and Integrative Sciences, Jawaharlal Nehru University, New Delhi, 110067 India; 2 Finance and Accounting area, Indian Institute of Management, Vastrapur, Ahmedabad, 380015 India; 3 Economics area, Indian Institute of Management, Vastrapur, Ahmedabad, 380015 India

## Abstract

We demonstrate the existence of an empirical linkage between nominal financial networks and the underlying economic fundamentals, across countries. We construct the nominal return correlation networks from daily data to encapsulate sector-level dynamics and infer the relative importance of the sectors in the nominal network through measures of centrality and clustering algorithms. Eigenvector centrality robustly identifies the backbone of the minimum spanning tree defined on the return networks as well as the primary cluster in the multidimensional scaling map. We show that the sectors that are relatively large in size, defined with three metrics, viz., market capitalization, revenue and number of employees, constitute the core of the return networks, whereas the periphery is mostly populated by relatively smaller sectors. Therefore, sector-level nominal return dynamics are anchored to the real size effect, which ultimately shapes the optimal portfolios for risk management. Our results are reasonably robust across 27 countries of varying degrees of prosperity and across periods of market turbulence (2008–09) as well as periods of relative calmness (2012–13 and 2015–16).

## Introduction

The structure of a complex network comprising dissimilar units of a system interacting with each other, affects the dynamics of the system and provides deeper insight about the functionality of the system, its evolution, and the role of individual or modular units. Thus, network analysis has become a primary tool in the fields as diverse as systems biology, ecology, epidemiology, sociology, economics and finance^1^. A modern economy can be pictured as a network, comprising multiple sectors specialized for producing different sets of goods and services, which exhibit high degree of inter-connectedness arising out of the dynamic nature of the underlying system. There are two levels of connectedness that we observe across sectors. At the nominal level, the fluctuations of returns from the sectoral indices show the degree of return co-movements across sectors. At the production level, the flow of goods and services across sectors gives rise to dispersion in relative sizes of these sectors.

Widespread existence of bubbles in financial markets and extreme movements of return series indicate that the relationship between macroeconomic fundamentals and the asset prices is unstable^2^. The ‘excess volatility puzzle’ in the stock markets refers precisely to this disconnect between volatility of asset returns and movements of the underlying fundamentals^3^. Recent research emphasizes the roles played by bounded rationality as being important causal factors for the observed disconnect^4^. In this paper, we present an alternative view that the co-movements in financial assets are anchored to the corresponding macroeconomic fundamentals. Thus, nominal returns from individual assets might drift far from what can be predicted using expected cash-flow, while the joint evolution of the co-movements of returns are still related to aggregate size variables like market capitalization, revenue or number of employees.

In the following, we study the economy at the mesoscopic level. There are some well known regularities at different levels of granularity. At the micro level, firm size distributions show power law decays^5^ and bi-exponential growth size distributions^6^. Existence of a scaling relationship between size of the firms and the corresponding volatility is also known^6^. At the macro level, similar features are seen, for example, between country size and volatility^7^. These suggest that there might be universal features of growth processes of economic entities (see also Lee *et al*.^8^). Gabaix^7^ further argued that the dispersion in relative sizes of firms contributes substantially to the aggregate volatility of an economy, providing a link from the micro level to the macro level. A complementary view has emerged from the network literature that the dynamics at the intermediate sectoral level could play an important role in shaping the aggregate macro-level dynamics^9^. The economy at the *meso* level identifies with the aggregate production process, while being granular enough to capture the network structure of co-movements in return fluctuations across sectors.

To study the topology of the return correlation network, we construct correlation matrices from sectoral indices for 27 countries, and apply two commonly used clustering algorithms, viz., minimum spanning tree (MST hereafter) and multi-dimensional scaling (MDS hereafter), to group sectors based on their co-movements. The influence of the sectors in the whole network can be found by using eigenvector centrality, which is able to handle both directed as well as weighted graphs^10^. In this paper, we propose a method to find a binary characterization of the ‘core-periphery’ structure by using a modification of the eigenvector centrality. Such classification of the sectors according to whether they belong to the core or the periphery, allows us to pin down exactly which sectors are driving the market correlations. We show that those sectors identified as core by the centrality measure, also constitute the backbone of the MST and cluster very closely in the MDS maps, thereby confirming the robustness of our method of extraction of the core-periphery structure.

To establish the connection between the financial network and the underlying production process, we regress the eigenvector centrality measure on sector sizes defined with three different metrics, viz., market capitalization, revenue and employment, all aggregated at the sectoral level. Market capitalization is our primary variable of size as this has the most extensive coverage across countries and time. The results across 27 countries clearly indicate that the dispersion in economic size explains the variation in the dispersion of sectoral centralities in the return correlation matrix. This is the primary finding of our paper, as it establishes the linkage between the economic fundamentals and the fluctuations of the return series.

Finally, we study the risk diversification of a portfolio comprising sectoral indices, based on the eigenvector centralities. For the sake of simplicity, we use a rudimentary Markowitz portfolio allocation problem and show that the core sectors, i.e., the ones with sufficiently high centralities, do not usually appear in a minimum variance portfolio. Intuitively, very large sectors contribute significantly to the movement of the return correlations and they constitute the ‘market factor’ of correlations. Hence, for reduction of the volatility of the portfolio, the weights assigned to such sectors contributing to the aggregate risk are necessarily minimized.

We perform statistical tests on a comprehensive list of 27 countries that includes developed as well as developing countries across five continents, totaling 65 sectors in the financial economies. We base our analyses on a recent and relatively calm period (2015–16), an intermediate period (2012–13) and then compare and contrast with a volatile period (2008–09), in order to check the robustness of our findings across time. We show that the 2015–16 and 2012–13 periods give very consistent results (25 out of 27 countries are in expected directions); 2008–09 period is also largely consistent (22 out of 26), although there are some aberrations, as the number of statistically insignificant relationships increases.

## Results

### Core-periphery Structure and Sectoral Dynamics

**Figure 1:**
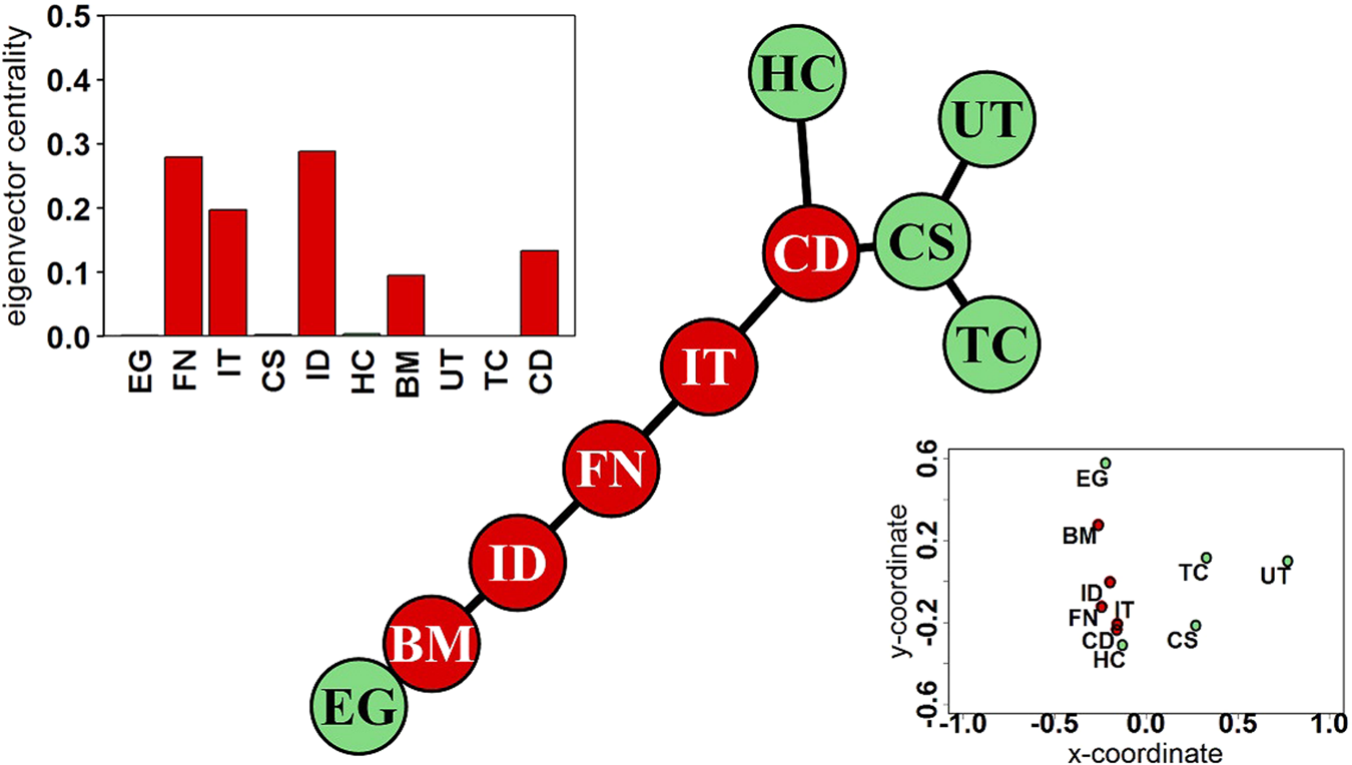
Results for USA: Identification of the sectors that are in the core (red) and periphery (pale green) of the minimum spanning tree, where the nodes represent different sectors; sectoral abbreviations given in the Table 1 in Materials and Methods. *Left Inset*: Eigenvector centralities of *ρ* ^32^. *Right Inset*: Multidimensional scaling, where the different sectors are plotted as coordinates in a map.

Given the return correlation matrix ***ρ***, we computed the modified eigenvector centralities to find the core sectors of the countries. To visualize the co-movements and clusters of sectors based on return correlations, we applied two clustering algorithms, viz., MDS and MST. Figure 1 shows the MST; Fig. 1 *Left Inset* shows that using the eigenvector centrality, we can identify that out of 10 sectors of the USA, 5 sectors constitute the core of the economy, viz., Finance (FN), Information Technology (IT), Industries (ID), Basic Materials (BM) and Consumer Discretionaries (CD) (see Table 1 in Materials and Methods for names of the sectors); Fig. 1 *Right Inset* shows the MDS. The MST generates a core-periphery structure based on minimizing the distance between correlated sectors, and since it is a hierarchical clustering method, similar sectors can be found close to each other (or in one branch). Similarly, closer the sectors are placed on the MDS map, more correlated (similar) they are; farther they are placed on the map, less correlated they are.

**Table 1:** Abbreviations of the 65 sectors analyzed.

Labels	Sectors	Labels	Sectors	Labels	Sectors
**AF**	Agro & Food Industry	**EM**	Electrical Machinery	**OG**	Oil and Gas
**AG**	Agriculture	**EU**	Energy & Utilities	**PC**	Property & Construction
**AM**	Automobiles	**FB**	Food & Beverages	**PE**	Power & Energy
**BC**	Building & Construction	**FN**	Finance	**PG**	Personal Goods
**BF**	Banks & Finance	**GD**	Gold	**PH**	Petrochemical
**BFT**	Beverage, Food & Tobacco	**HC**	Health Care	**PL**	Plantation
**BK**	Bank	**HG**	Household Goods	**PR**	Property
**BM**	Basic Materials	**HT**	Hotel & Tourism	**PSU**	Public Sector Undertaking
**BR**	Basic Resources	**ID**	Industries	**RB**	Rubber
**CC**	Consumer & Cyclical	**IF**	Infrastructure	**RE**	Real Estate
**CD**	Consumer Discretionary	**IP**	Industrial Production	**RT**	Retail
**CD1**	Consumer Durables	**IS**	Insurance	**RY**	Realty
**CE**	Cement	**IT**	Information Technology	**SU**	Securities
**CG**	Consumer Goods	**MF**	Manufacturing	**TC**	Telecom
**CG1**	Capital Goods	**MG**	Mining	**TD**	Trade
**CH**	Chemicals	**MI**	Multi Investments	**TE**	Transport & Equipment
**CM**	Construction & Materials	**MID**	Miscellaneous Industries	**TP**	Transport
**CN**	Construction	**MO**	Mining & Oil	**TS**	Trade & Services
**CP**	Consumer Products	**MOT**	Motors	**TX**	Textiles
**CS**	Consumer Staples	**MP**	Metal Products	**UT**	Utilities
**CSR**	Consumer Services	**MP1**	Media & Publishing	**WS**	Wholesale
**EG**	Energy	**OC**	Oil & Coal Products

**Figure 2:**
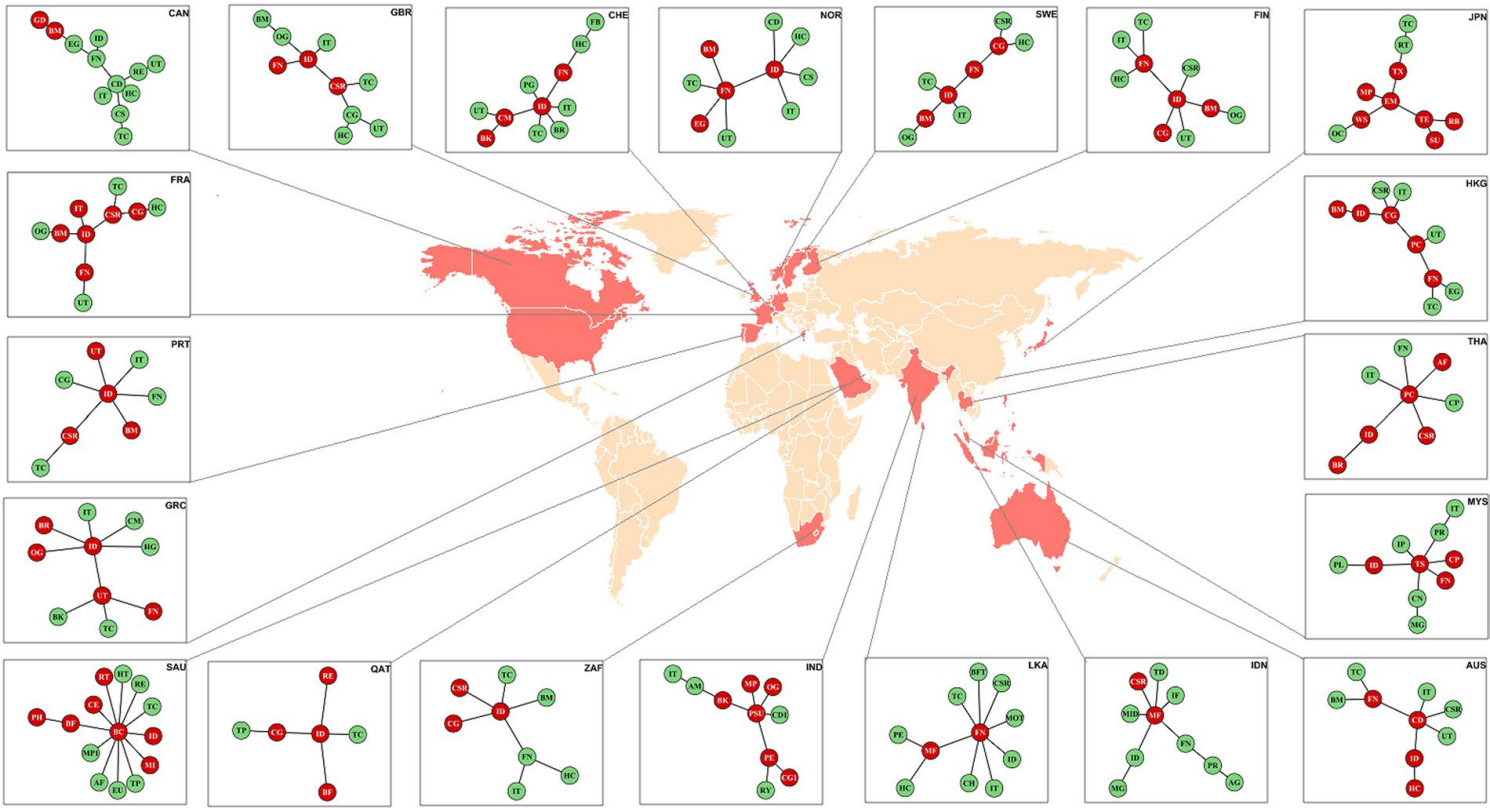
Minimum spanning trees for 20 countries out of the 27 countries (shown in peach) that are being studied across the globe. The core sectors are colored red (darker shade), while the sectors in the periphery are in pale green (lighter shade); sectoral abbreviations given in the Table 1 in Sec. Materials and Methods. The world map has been generated with the open source software R-Statistical Computing and Graphics (version 3.3.2 (2016-10-31)), using the in-built library(rworldmap).

There are two major observations: First, MST shows that all core sectors form a chain or the “backbone” in the tree. Similarly, the MDS also reiterates most of the information: the core sectors, as identified by the modified eigenvectors centrality, belong to one cluster in the MDS; all sectors with negligible centrality are spaced in the periphery in the MDS. Thus, our method of the modified centrality to extract the core sectors is reinforced by the clustering algorithms, indicating the robustness of our findings. Second, the MST built from the return correlation matrix, contains information about the actual production structure of the economy. For example, Energy (EG) is most closely related to Basic Materials (BM), which in turn is related to Industries (ID), and so on. On the other end of the MST, Consumer Staples (CS) is connected to Telecom (TC) sector, Utilities (UT) and Consumer Discretionary (CD). Again, this qualitative feature is quite robust, as observed in almost all the countries analyzed. In Fig. 2, we present similar MSTs (with the core/backbone colored in red) for 20 other countries, elucidating the core-periphery structures. We complement the analysis with subgraph centrality measure^11^ as well as usage of disparity filter^12^ to extract the backbone of the networks (see Results in Supplementary Information).

**Figure 3:**
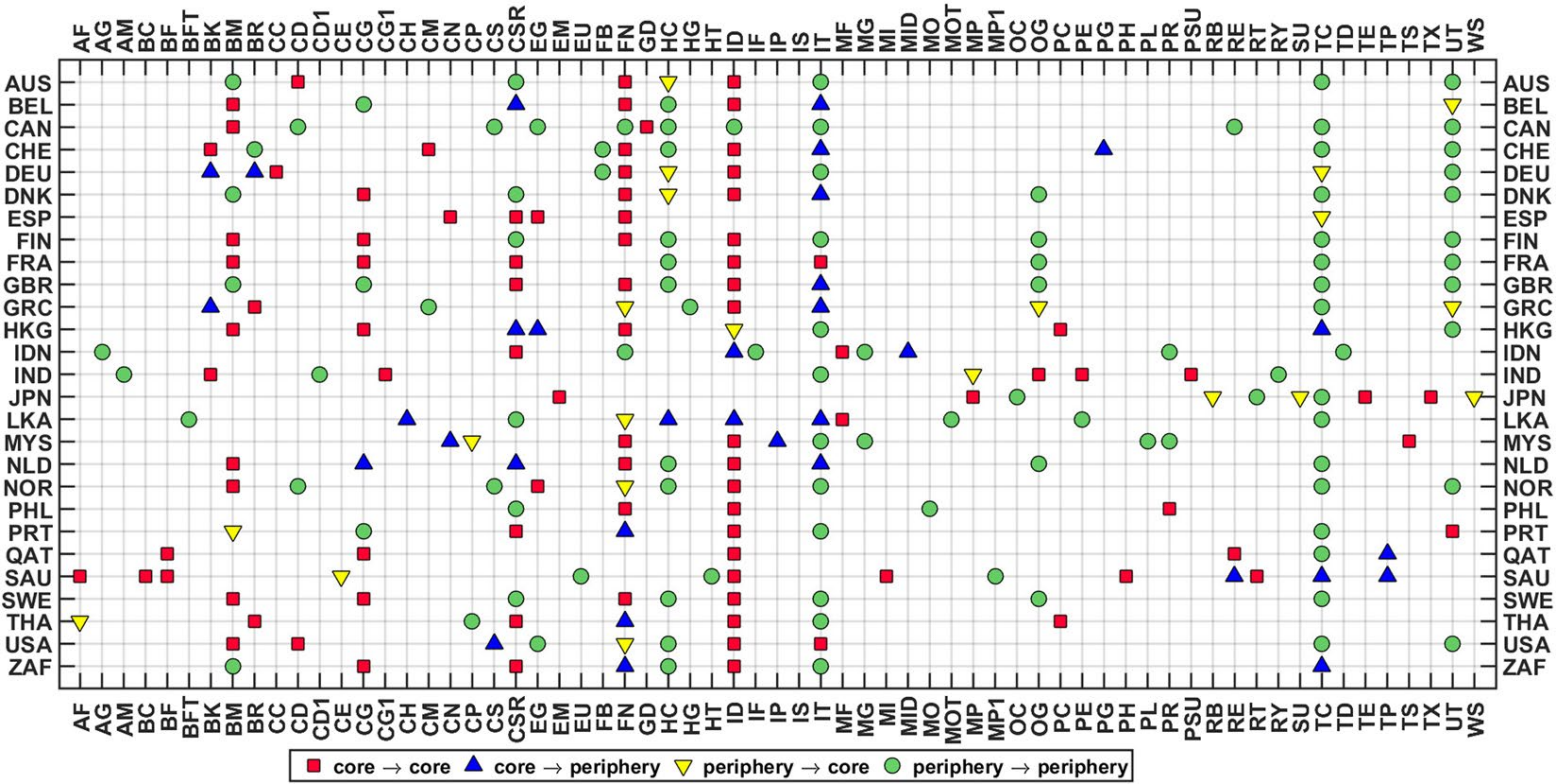
Sectoral dynamics and core-periphery structure: Chart of the 65 sectors (horizontal axis) in the 27 countries (vertical axis), showing the evolution of the core-periphery structure over two snapshots, 2008–09 and 2015–16. *Interpretation: x* → *y* implies *x* in 2008–09 became *y* in 2015–16 (*x*, *y* ∈ {*core*, *periphery*}). Visual inspection reveals that Finance (FN) and Industries (ID) sectors are frequently occurring in the core across almost all countries. Sectoral abbreviations given in the Table 1 in Materials and Methods. Also, countries like Canada (CAN), Finland (FIN), France (FRA), Philippines (PHL) and Sweden (SWE) maintain a stable core-periphery structure with no change, whereas the rest of the countries show different degrees of change in the core-periphery structure.

**Figure 4:**
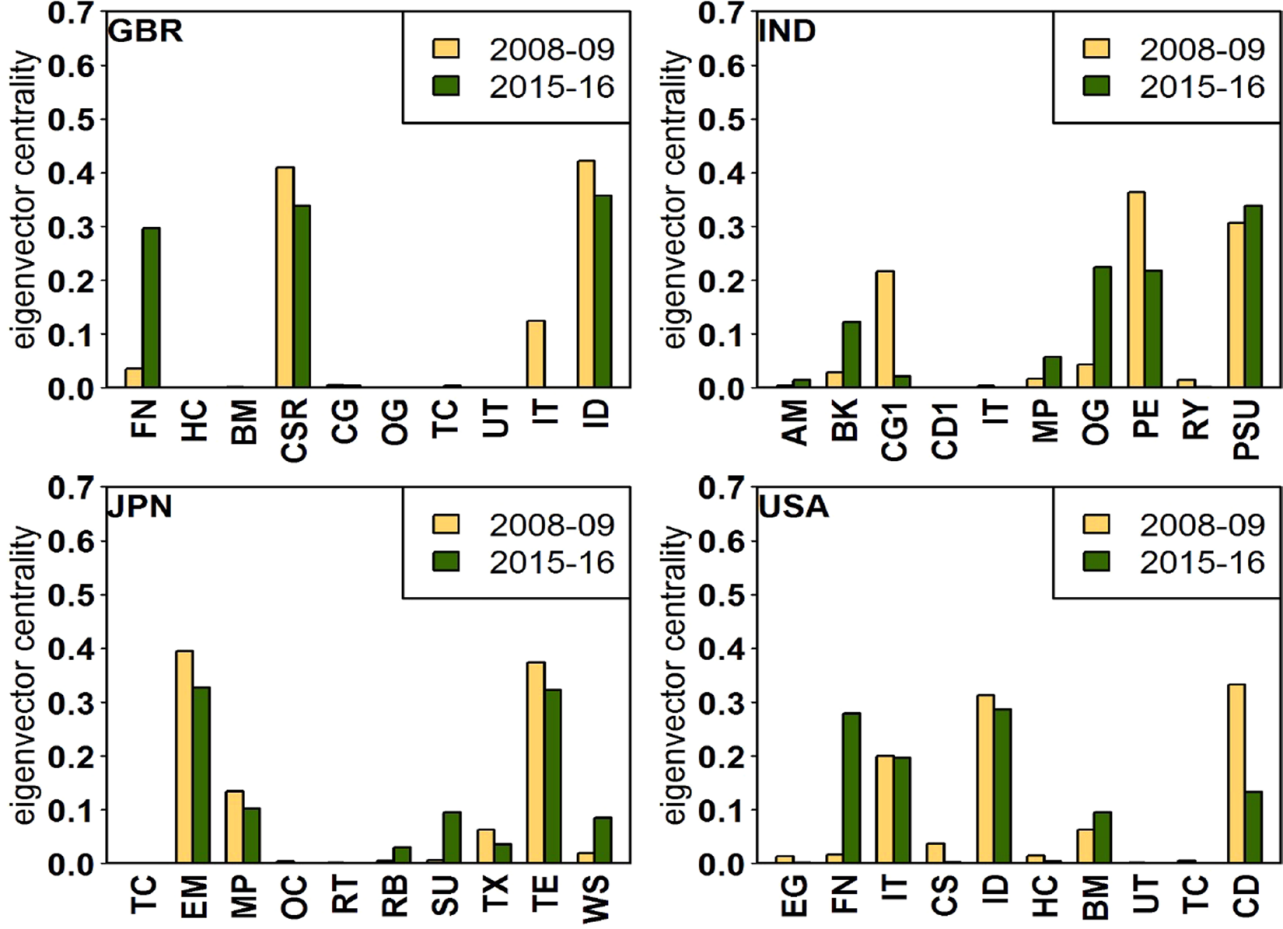
Sectoral dynamics and robustness: The comparison of the eigenvector centralities for the years 2008–09 (light orange) and 2015–16 (dark green) for four countries. *Upper Left*: United Kingdom (GBR), *Upper Right*: India (IND), *Lower Left*: Japan (JPN), *Lower Right*: United States of America (USA).

Figure 3 shows the sectoral dynamics and the core-periphery structure for all countries. As can be seen, there are at least two sectors in the core for all countries, but the core-periphery structure often changes with time (when compared for the periods 2008–09 and 2015–16). Figure 4 shows the comparison among the modified eigenvector centralities for the years 2008–09 and 2015–16, for the four countries: United Kingdom, India, Japan, and United States of America, as examples. The relative importance of each sector can be compared for the volatile and calm period. Certainly, the sectoral dynamics are interesting to note in the different countries, and may help in taking important policy decisions in economic growth and development. Here, we note that the above discussion is based on the core-periphery distinction arising out of the eigenvector centralities (see Materials and Methods). To check robustness, we have also computed the core-periphery structure by using the disparity filter algorithm developed by Serrano *et al*.^12^. All results can be found in the Supplementary Information and predictions of that algorithm matches to a large extent with our findings.

### Financial Fluctuations and Economic Fundamentals

Most importantly, we now show that the core-periphery structure based on the return correlation matrix, ***ρ***, has a stable relationship with the relative sizes of the sectors. We study the variations in the eigenvector centralities of the return correlation matrix, and exploit the variations in three major variables, viz., aggregate market capitalization, aggregate revenue and aggregate employment. We have described how we constructed the sector-level data by aggregating the company-level data corresponding to each sector in each country, in the Materials and Methods section. For finding the effect of size on centrality, we execute the following regression,

1 $$centralit{y}_{i}={\beta }_{0}+{\beta }_{1}siz{e}_{i}+{\varepsilon }_{i},$$

for three snapshots covering the last decade (2008–09, 2012–13 and 2015–16, see Supplementary Information), where *β*_0_ and *β*_1_ are constant terms and *ε*_*i*_ denotes error terms. The ordinary least squares method minimizes the sum of the squared errors, to estimate the coefficients *β*_0_ and *β*_1_. Throughout our regression analyses, we have used standardized variables: ([variable − mean(variable)]/standard deviation(variable)) so that we can compare estimated *β*_1_ for different size variables. We have used eigenvector centrality in our basic framework to estimate Eq. 1. To check robustness of our findings, we have also estimated subgraph centrality^11^. The results are consistent with the estimates done with eigenvector centrality (see Supplementary Information).

**Figure 5:**
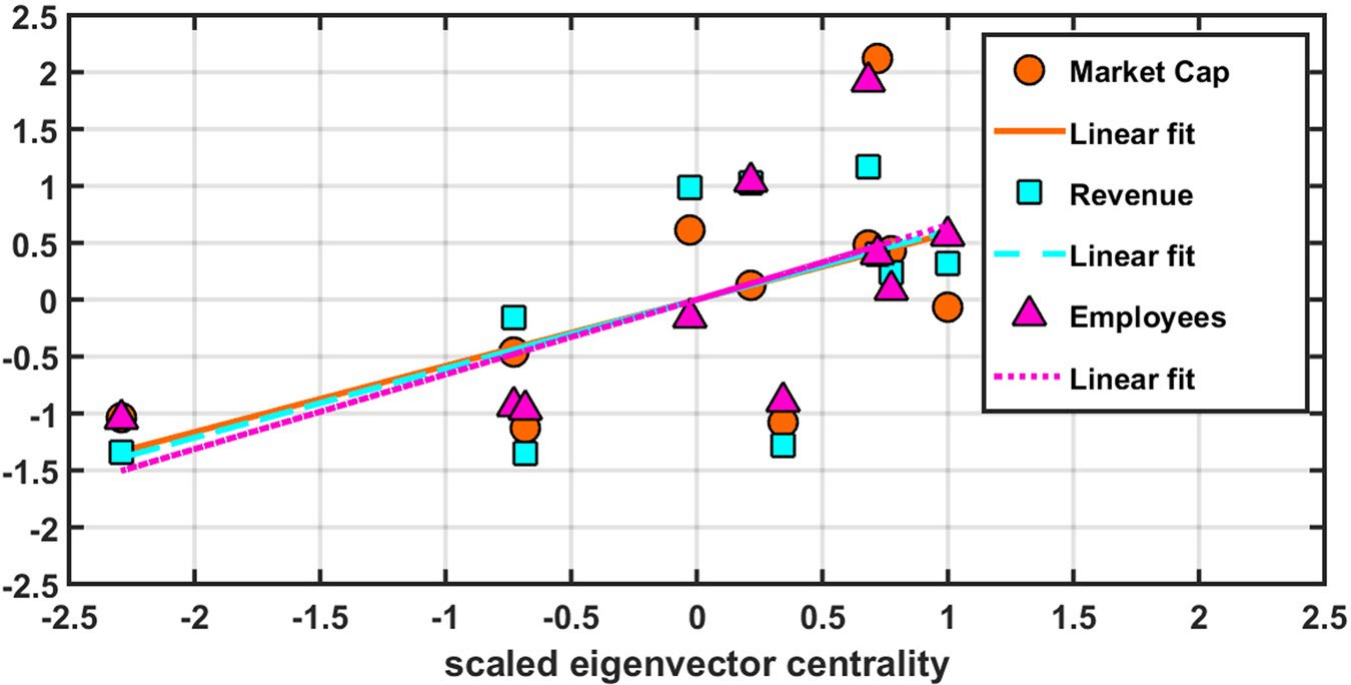
Results for USA: Linear regressions of scaled eigenvector centrality with scaled market capitalization (orange filled circles), scaled revenue (cyan filled squares), and scaled number of employees (magenta filled up-triangles). The best fits (linear regressions) are plotted as lines for market capitalization (orange solid), revenue (cyan long dashed) and employees (magenta short dashed).

**Figure 6:**
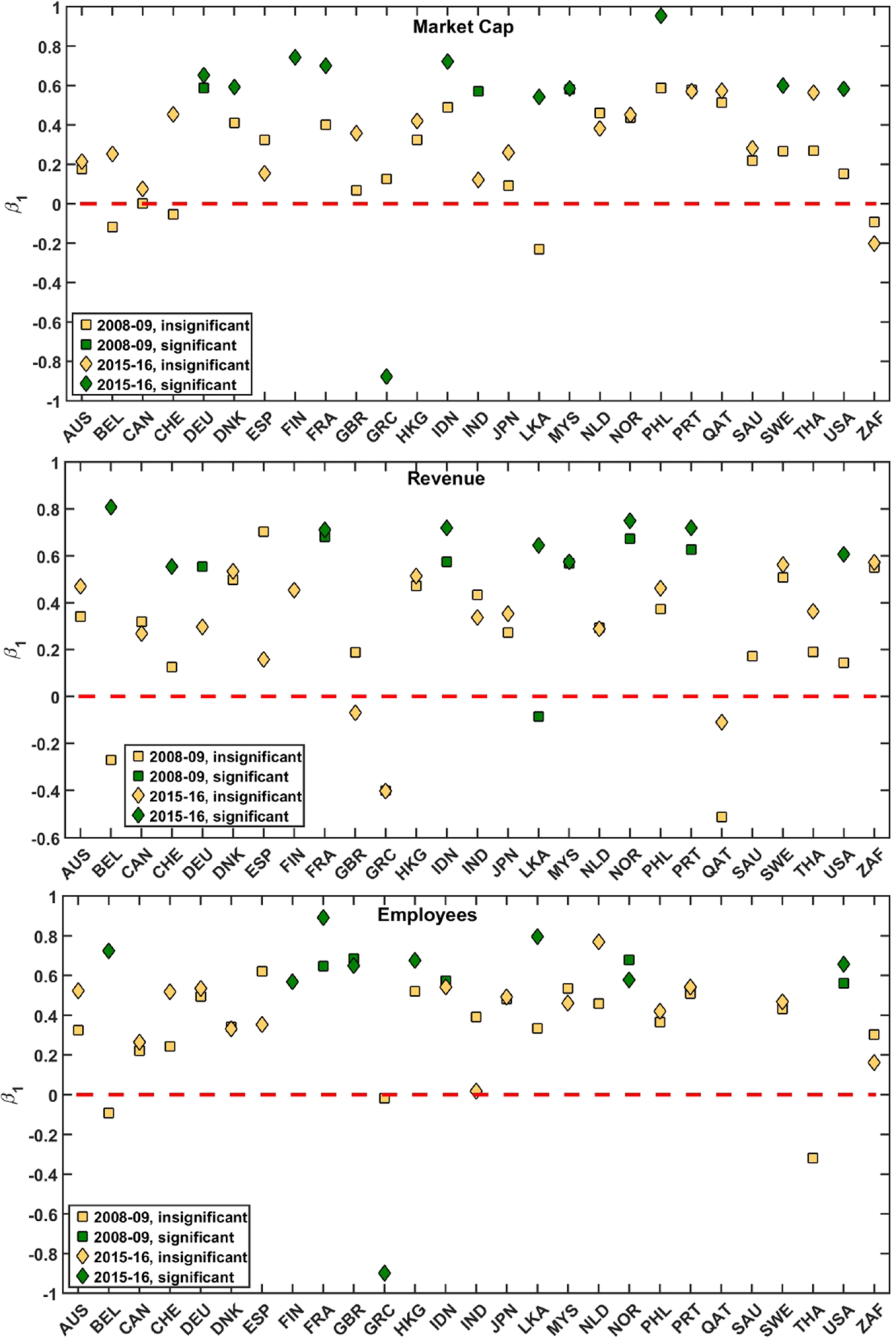
Comparison of the regression results (estimates of *β*_1_ using Eq. 1) to explain variation in the sectoral eigenvector centralities by the variation in sector-level macro data. *Upper*: market capitalization, *Middle*: Revenue, *Lower*: Employees, for the years 2008–09 and 2015–16. Detailed estimation results are given in the Supplementary Information Tables S2–S10.

In Fig. 5, we plot the linear regressions of scaled eigenvector centrality with the (scaled) market capitalization, revenue and employees for the USA. We have performed similar analyses for the other countries, and tabulated the results in the Supplementary Information Tables S2–S10, which suggest that generally, such a mapping exists for almost all countries. Figure 6 shows the results of regressing the sectoral eigenvector centralities on the sector-level aggregate market capitalization, revenue, and employees, for the years 2008–09 and 2015–16. As we see in the *Upper* panel, for 2015–16, the estimated coefficients *β*_1_ for market capitalization are positive for 25 out of 27 countries (11 of them are statistically significant at 10%). The two countries which have very mildly negative relationships, are Greece (significant) and South Africa (insignificant). We have not plotted 2012–13 data in order to keep the figure clean. As can be checked from the Supplementary Information, estimated *β*_1_ is positive for 25 out of 27 countries (7 of them are siginificant at 10%). For 2008–09, estimated *β*_1_ is positive for 22 out of 26 countries (3 of them are significant at 10%). Belgium, Switzerland, South Africa and Sri Lanka have negative relationships. The *Middle* panel shows that in 2015–16, the estimated coefficient *β*_1_ for revenue is positive for 23 out of 26 countries (9 are significant at 10%). The three countries which have negative (and statistically insignificant) relationships, are Greece, Qatar and United Kingdom. For 2008–09, the coefficient *β*_1_ is positive for 22 out of 26 countries (9 are significant at 10%). Finally, the *Lower* panel shows that for 2015–16, *β*_1_ for employees was positive for 23 out of 24 countries (9 are significant at 10%) The only country which has negative (and statistically significant) relationship, is Greece. For 2008–09, the coefficient *β*_1_ is positive for 21 out of 24 countries (5 are significant at 10%). To check robustness of our results, we have also computed subgraph centrality following Estrada *et al*.^11^. The regression results can be found in the Supplementary Information (Tables S11–S19) and the results are very close to the ones computed with eigenvector centrality. Finally, we present another robustness check with degree centrality or *node strength*, which is a first order approximation of eigenvector centrality (see Materials and Methods for an explanation as to why node strength for a weighted network approximates the eigenvector centrality), being positively related to size variables. Regression results have been given in the Supplementary Information (Tables S20–S28). All results, by and large, corroborate our main observations and findings stated above. Thus combining all modifications of the centrality measure in the basic regression model, we find that eigenvector centrality is a robust specification to model the financial network.

There is already an existing finding that centralities in input-output networks are closely related to the relative sizes of the corresponding nodes (see Acemoglu *et al*.^9^). However, our finding is complementary in nature because here we show that the centralities based on nominal return fluctuations can be directly mapped to the relative size, i.e., the return network is also very closely related to the underlying size effects. An immediate corollary is that the core sectors of the return correlation network are also economically big, and hence, the market effect of the correlations are driven by those sectors which have very high market capitalization (or other indicators like revenue and employment). It can also be easily verified that the correlation between a sector’s size and its average co-movement with other sectors in the financial market is also high (see Results in Supplementary Information Tables S29–S31). Next, we discuss the relationship between the eigenvector centralities across sectors and the riskiness of a portfolio.

### Constructing the Minimum Risk Portfolio

**Figure 7:**
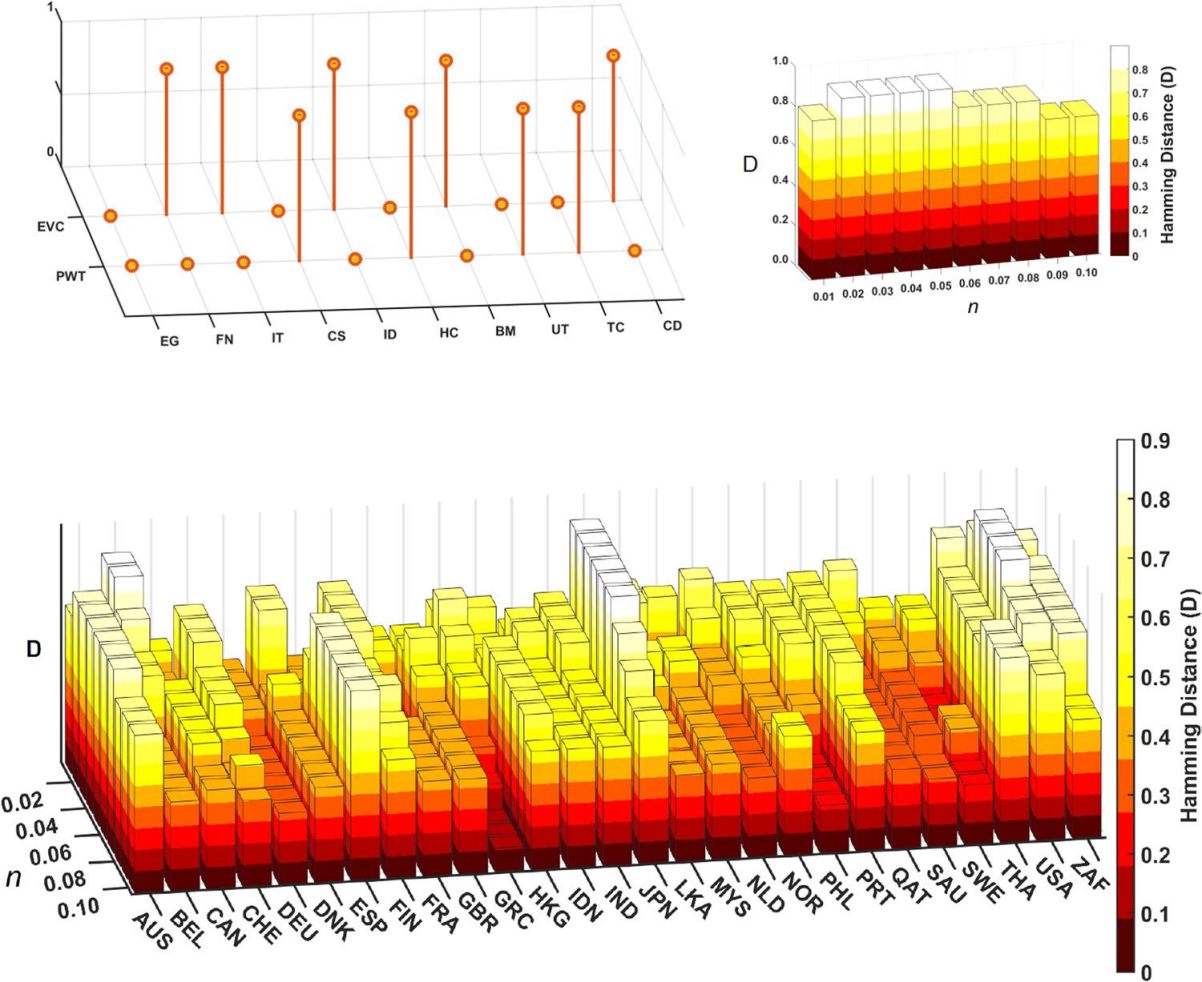
*Upper Left*: Relationship between the bit-strings of sectoral centralities (EVC) and their corresponding inclusion in the portfolio (PWT) for the different sectors of the USA. The threshold values *θ*_*e*_ and *θ*_*p*_ (as 2% of the coefficient of variations of EVC and PWT, respectively) would determine, whether the sector is central or not (EVC is 0 or 1), or whether the corresponding sector would appear in the optimal portfolio or not (PWT is 0 or 1). *Upper Right*: The Hamming distance *D* computed from the bit-strings EVC and PWT for USA, against the different values of *n* (percentage) of the coefficient of variations of EVC and PWT, respectively, which determine the threshold values *θ*_*e*_ and *θ*_*p*_. *Lower*: The Hamming distance *D* computed from the bit-strings EVC and PWT, against the different values of *n* (percentage), for the different countries, plotted as a 3D-bar.

In this part, we study how the sectoral centralities influence the aggregate risk of a portfolio. For the purpose of simple exposition, we compute the benchmark model of Markowitz portfolio selection with the sectoral return data. Assuming rational investors with risk-aversion, the investors will minimize

2 $$w^{\prime} {\rm{\Sigma }}w-{\rm{\Theta }}R^{\prime} w,$$

with respect to the weight vector *w*, where Σ is the covariance matrix of the sectoral returns, *R*′ is the expected return vector, and Θ is a parameter, which denotes the risk appetite of the investor. We set a short-selling constraint (*w*_*i*_ ≥ 0) and Θ equal to zero for finding the minimal risk portfolio, which will entail a convex combination of sectoral returns (the other extreme would lead to a corner solution). Our main observation is that the optimal weight vector, *w* ^*^, is negatively related to the eigenvector centralities, i.e., if a sector is very “central” in the return correlation network, then it is less likely to appear in the optimal portfolio with minimum risk (and no short selling). We demonstrate this in a naive way for each country: We construct threshold values *θ*_*e*_ and *θ*_*p*_, as a fixed percentage (say *n*%) of the coefficient of variation (standard deviation/mean), for both the eigenvector centralities as well as the minimum risk portfolio weights. These threshold values *θ*_*e*_ and *θ*_*p*_ would determine, respectively, whether the sector is central or not (i.e., 0 or 1), or whether the corresponding sector would appear in your optimal portfolio or not (i.e., 0 or 1). So, for the vector of sectors, we would have two strings of 0’s or 1’s corresponding to the centrality vector (EVC) and the optimal weight vector (PWT), respectively. The Hamming distance *D* between any two bit-strings of equal length, is the number of positions at which the corresponding bits are different. So, the Hamming distance between the two strings EVC and PWT would tell how important the observation is for a particular country; the higher the value of *D*, the better the conformity. The sector which is central (i.e., 1) would not appear in your portfolio (i.e., 0), and so for any country the ideal finding would be that *D* is unity. The choice of the threshold(s), *θ*_*e*_ and *θ*_*p*_, equaled by the percentage(s) (*n*) of the coefficient of variation(s) in the vectors EVC and PWT, would be important for determining the Hamming distance *D* between the strings for any country (see Fig. 7 (*Upper*) for the USA). We can optimize the value of *D* against the percentage *n*, for all the countries, as shown in Fig. 7 (*Lower*). We found that *n* = 2, i.e., 2% was an optimal threshold value *θ*_*e*_ for most countries, which we then used to distinguish between the core and periphery sectors. Combined with the finding that core sectors in the return correlation network are bigger in size, the above finding implies that peripheral sectors contribute to lower the risk of a diversified portfolio.

## Summary and Conclusion

In this paper, we have analyzed financial and economic data for 27 countries at the sector level and provided a methodology to extract the core-periphery structure of the correlation networks in a binary fashion. The resulting generic rule of thumb was that economic size explained correlations across financial assets. We attribute significant importance to this finding as it provides a way to exactly pin down the sectors which are main drivers of financial fluctuations through the size effect. The way return series are constructed from price data by log differencing, the size differential of the prices across the sectoral indices should disappear due to the normalization. It is known that volatility of variables like firm-growth rates has a scaling relationship with size^6^. In a comparable context, Eisler *et al*.^13^ had shown that persistence of financial time series can be positively correlated to market capitalization. However, both of such features are specific to one time series only. The contribution of this paper is to show that even dispersion in co-movements across financial time-series of multiple sectors can also be explained by the underlying size factors.

The fact that the co-movements are still tied to the fundamentals after normalization by the average size is therefore intriguing. Our results suggest this finding is considerably robust across countries. An illuminating exception is Greece, which shows an exactly opposite relationship, perhaps due to its weak economic fundamentals along with severe crises in the financial markets in the recent times. In both volatile and calm periods, economically large (either in terms of market capitalization or revenue or employment) sectors in Greece are at the periphery of the return correlation networks, which constitutes an inverted relationship between the economy and the financial networks.

We showed that the variation in centrality in the return correlation matrix across sectoral indices, can be explained by the size dispersion across the sectors. This finding indicates that financial fluctuations are mapped to the macroeconomic fundamentals. From the perspective of portfolio optimization, we showed that the very big sectors that are also highly central in the return network, rarely appear in a risk-minimizing portfolio. Essentially, such sectors constitute the main drivers of market-wide fluctuations. In summary, our study sheds light on: (a) the mapping between the joint evolution of the financial variables and the underlying macroeconomic fundamentals, and (b) extracting information about the individual influences on aggregate risk from sector-level, disaggregated time-series data.

We have also shown that the relative importance of the sectors may change substantially over time although some sectors like finance and industry are at the core of a large fraction of countries. In general, our results indicate that the core may not be very stable. Possible reasons could be sectoral competition in terms of productivity and innovation and the resultant evolution^14^. The emergence of the core-periphery structure changes the complexity of the financial markets and has implications of the pricing of risk in the economy^15^. Our work indicates the advantages of using a binary characterization to reduce the computational burden by introducing proper identification of the country-specific core sectors, as opposed to considering the full network.

To conclude, we note that the recent applications of network theory in the macroeconomics literature has focused significantly on studying the dynamics of real economic quantities^16^, whereas the relevant finance literature has focused on the dynamics of nominal quantities^17–19^. The present work may provide a linkage between the above two. Thus, we make the point that the oft-quoted quips ‘too-big-to-fail’^20^ and ‘too-interconnected-to-fail’^21^ may not be as different as are currently thought of.

## Materials and Methods

### Data Description

We have used the sectoral price indices from the Thomson Reuters Eikon database^22^, within the time frames January 2008– December 2009, October 2012– September 2013 and October 2014– September 2016. We have analyzed the data for a total of 65 sectors (see Table 1 for sectoral abbreviations) for the following countries: (1) **AUS**- Australia (2) **BEL**- Belgium (3) **CAN**- Canada (4) **CHE**- Switzerland (5) **DEU**- Germany (6) **DNK**- Denmark (7) **ESP**- Spain (8) **FIN**- Finland (9) **FRA**- France (10) **GBR**- United Kingdom (11) **GRC**- Greece (12) **HKG**- Hong Kong (13) **IDN**- Indonesia (14) **IND**- India (15) **JPN**- Japan (16) **LKA**- Sri Lanka (17) **MYS**- Malaysia (18) **NLD**- the Netherlands (19) **NOR**- Norway (20) **PHL**- Philippines (21) **PRT**- Portugal (22) **QAT**- Qatar (23) **SAU**- Saudi Arabia (24) **SWE**- Sweden (25) **THA**- Thailand (26) **USA**- United States of America and (27) **ZAF**- South Africa, spread across the continents of the Americas, Europe, Africa, Asia and Australia. The time series data on the real variables, such as market capitalization, revenue and the number of employees within each sector, are also available in the same database although at the company level rather than at the sectoral level. Hence, for our purposes of constructing sector-level macro aggregate variables, we collected the companies listed within each sector for one particular country, and then aggregated the relevant company-specific variables across all such companies within the corresponding sector. We find that the US economy is a good representative of the empirical results. Note that due to the extensive requirement of data coverage to carry out the analysis, data for a few countries were not available in some periods (see Supplementary Information). However, such missing data are quite sparse.

### Correlation Coefficient and the Distance Metric

Returns series are constructed as *r*_*i*_(*τ*) = ln*P*_*i*_(*τ*) − ln*P*_*i*_(*τ* − 1), where *P*_*i*_(*τ*) is the adjusted closure price of sector *i* in day *τ*. Then the equal time Pearson correlation coefficients between sectors *i* and *j* is defined as *ρ*_*ij*_ = (〈*r*_*i*_ *r*_*j*_〉 − 〈*r*_*i*_〉〈*r*_*j*_〉)/*σ*_*i*_ *σ*_*j*_, where 〈...〉 represents the expectation and *σ*_*k*_ represents standard deviation of the *k*-th sector. We use ***ρ*** to denote the return correlation matrix.

We construct the distance matrix from the correlation coefficients using the following transformation, ${d}_{ij}=\sqrt{\mathrm{2(1}-{\rho }_{ij})}$, where 2 ≥ *d*_*ij*_ ≥ 0. All elements of the matrix *d*_*ij*_ are “ultrametric”^23–25^ and ***d*** to denote the distance matrix.

### Eigenvector Centrality

To analyze the influence of a sector in the whole network, the ranking of the sectors is measured by eigenvector centrality. It is not necessary that a sector with high eigenvector centrality is highly linked, for the sector might have few but important links. Given a matrix ***A***_*N*×*N*_, the eigenvector centrality is defined as a vector ***x***_*N*×1_, which solves

3 $${\boldsymbol{Ax}}={\lambda }_{m}{\boldsymbol{x}},$$

where *λ*_*m*_ is the dominant eigenvalue of ***A***.

In general, almost all pair-wise correlations are positive. However, in rare cases (e.g., the Gold sector in Canada), certain sectors show mild negative correlations with other sectors. To account for those cases, we consider the absolute value of the correlation matrix |***ρ***| for computing the eigenvector centrality, since according to the *Perron-Frobenius* theorem, a real square matrix with positive entries has a unique largest real eigenvalue and the corresponding eigenvector has strictly positive components. Finally, we normalize the centrality vector ***x*** such that ∑_*i*_ *x*_*i*_ = 1.

We have also considered degree centrality or node strength, which is a first order approximation of eigenvector centrality, as a measure of influence in the financial network^1^. To see why degree centrality for a weighted network approximates the eigenvector centrality, let us imagine a Markov process given *x*_(*t*+1)_ = *Ax*_*t*_, where *A* denotes the transition matrix. If the process converges, then the solution solves *x*_∞_ = *Ax*_∞_ implying that the dominant eigenvector is the steady state solution. Note that from a network theoretic view, *x*_∞_ is also the eigenvector centrality for an adjacency matrix *A*. Now consider *x*_0_ being a vector of ones. Then *x*_1_ (=*Ax*_0_) is clearly the degree centrality. Thus degree centrality *x*_1_ is the first order approximation of the asymptotic result eigenvector centrality *x*_∞_. As we did for the eigenvector centrality, we normalize the node strength vector such that the sum across sectors is 1.

### Subgraph Centrality

To complement the analysis with eigenvector centrality, we also compute another centrality measure – subgraph centrality^11^. At the country level, one can also compute the Estrada index^26^, which is a topological index for a graph. Given a sequence of eigenvalues {*λ*_*j*_}_*j*∈*N*_, the Estrada index of a network *N* is defined as

4 $$EE(N)=\sum _{i=1}^{N}exp({\lambda }_{i}\mathrm{)}.$$

Let us denote the eigenvector associated with the *i*-th eigenvalue *λ*_*i*_ by *v*_*i*_ where *v*_*i*_ = [*v*_*i*_(1), …, *v*_*i*_(*N*)]. Then the subgraph centrality of the *j*-th node is defined as

5 $$SC(j)=\sum _{i=1}^{N}{v}_{i}{(j)}^{2}exp({\lambda }_{i}\mathrm{)}.$$

### Determining Core-Periphery Structure

To identify the core-periphery structure there are several filtering methods existing in the literature. All filters have limitations and one has to consider them in relation to the problem under analysis.

We first consider a modification of the centrality measure to identify the core-periphery structure in a binary fashion. We give a transformation to the correlation matrix such that the weak correlations acquire asymptotically zero weights and the strong correlations acquire enhanced weights, while maintaining positive signs. Hence, instead of the level values of the correlation coefficients, we consider ***ρ*** ^*c*^, where *c* is a sufficiently large *even* number. In our case *c* = 2^5^ = 32, the lowest value that gives reasonably good estimates of the backbone of the minimum spanning tree. We thus present results for *c* = 32 although, in principle, one can use higher values as well. To determine the core sectors of a country, we then construct a threshold value *θ*_*e*_, as a fixed percentage of the coefficient of variation (standard deviation/mean) for the eigenvector centralities. If the sectoral centrality is above the threshold value *θ*_*e*_, then the sector is considered core, otherwise not.

To provide a complementary picture to the proposed algorithm of the core-periphery structure with the eignvector centrality, we have also used an algorithm proposed by Serrano *et al*.^12^ to extract the backbone of the networks. The disparity filter algorithm extracts the network backbone by considering the relevant edges at all the scales present in the system and exploiting the local heterogeneity and local correlations among the weights. The disparity filter has a cut-off parameter *α*_*c*_, which determines the number of edges that are reduced in the original network. The filter however, preserves the cutoff of the degree distribution, the form of the weight distribution, and the clustering coefficient. These comparative results can be found in the Supplementary Information.

## Electronic supplementary material

Supplementary information
