# Supplementary material for: Financial fluctuations anchored to economic fundamentals: A mesoscopic network approach
Source: Sci Rep. 2017 Aug 14;7:8055. doi: 10.1038/s41598-017-07758-9 (PMC5556004; doi:10.1038/s41598-017-07758-9)
Supplement: Supplementary file 1 — Supplementary information [file 41598_2017_7758_MOESM1_ESM.pdf]

Financial fluctuations anchored to economic fundamentals: A mesoscopic network approach

Kiran Sharma[1], Balagopal Gopalakrishnan[2], Anindya S. Chakrabarti[3,*], and Anirban Chakraborti[1,]**

[1]School of Computational and Integrative Sciences, Jawaharlal Nehru University, New Delhi-110067, India.
[2]Finance and Accounting area, Indian Institute of Management, Vastrapur, Ahmedabad 380015, India.
[3]Economics area, Indian Institute of Management, Vastrapur, Ahmedabad 380015, India.
[*]anindyac@iima.ac.in
[**]anirban@jnu.ac.in

ABSTRACT

All Supplementary Information consisting figures, regression tables, etc. are provided.

Description

In this document, we provide all additional tables and figures supporting the main paper. Fig. 1 shows the coefficients for regressing subgraph centrality on size of the three explanatory variables, viz., market capitalization, revenue and number of employees. Fig. 2 shows the Estrada index computed for all countries over two time slots (2008-09 and 2015-16). Besides using the algorithm of the core-periphery structure with the eignvector centrality, we have also used the disparity filter proposed by Serrano et al. (2009), "Extracting the multiscale backbone of complex weighted networks", *Proceedings of the National Academy of Sciences*, **106**: 6483–6488. Table S1 shows the core-periphery structure of the economies with two different algorithms. Thus, the core sectors using the benchmark algorithm, based on eigenvector centralities, proposed in the current paper are presented in column 4, table S1. The second column of the table S1 contains the range of the threshold parameter, α_c, allowed by the network within which the algorithm returns result with interior solutions for different countries. Values of the threshold parameter below the lower limit of α_c admit the whole network as core and values of the threshold parameter above the upper limit of α_c returns null set as core. Thus, we have included the range for the parameter α_c between these two limits. We have chosen the "optimal" value by maximizing the number of matches between the core sectors predicted by the disparity filter and those predicted by eigenvector centrality. It is seen that these two are fairly close to each other, with exact matchings in 13 out of 27 cases.

Tables S2– S4 contain detailed results of regressing scaled eigenvector centrality on scaled size indices with 2015-16 data; market capitalization, revenue and number of employees respectively. Corresponding results for 2012-13 data can be found in tables S5– S7, respectively. Similarly, corresponding results for 2008-09 data can be found in tables S8– S10, respectively. We have also carried out the regressions using (i) subgraph centrality as the dependent variable – See the corresponding results in tables S11– S19, and (ii) node strength as the dependent variable – See the corresponding results in tables S20– S28. Finally, we present the average correlation between the size of sectors in a country and its average co-movement with the other sectors in the financial market. Tables S29– S31 show the results for 2015-16, 2012-13 and 2008-09, respectively.

Results

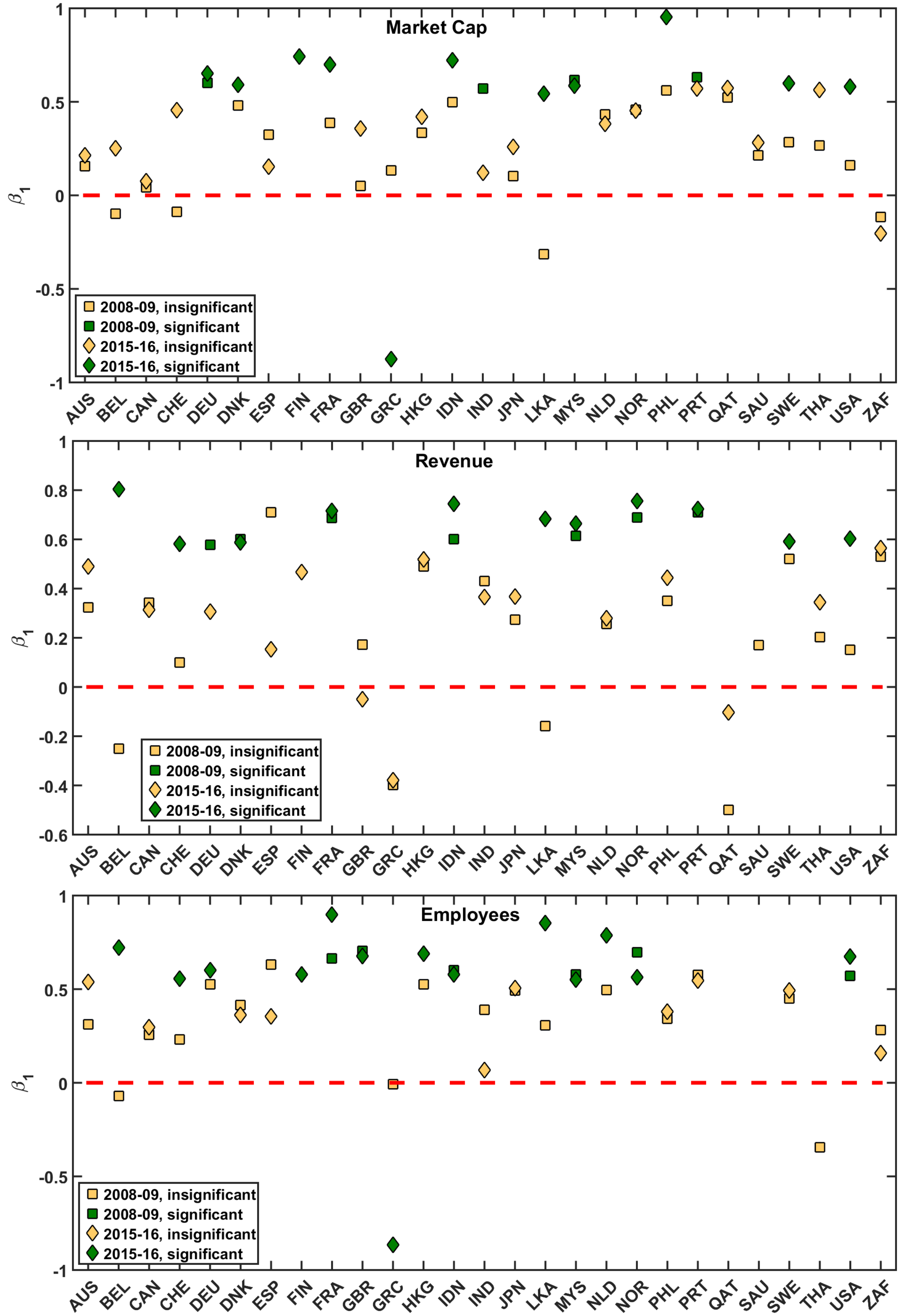

Figure 1. (Color online) Comparison of the regression results (estimates of β_1 using Eq. 1 in the main text) to explain variation in the sectoral subgraph centralities by the variation in sector-level macro data. *Upper*: market capitalization, *Middle*: revenue, *Lower*: employees, for the years 2008-09 and 2015-16. Detailed estimation results are given in tables S11– S13 for 2015-16 and in tables S17– S19 for 2008-09 data.

Table 1. Comparative analyses of the algorithms to determine the sectoral core-periphery structure: The disparity filter algorithm with cutoff parameter α_c, and our proposed method of the eignvector centrality along with threshold parameter θ_e.

Country	Range of α_c	Optimal α_c	Core using disparity filter	Core using eigenvector centrality
**Australia**	0.36-0.52	0.45	CD HC FN ID	CD HC FN ID
**Belgium**	0.33-0.50	0.40	CSR ID BM FN HC UT	ID BM FN UT
**Canada**	0.34-0.55	0.55	EG GD FN BM	GD BM
**Switzerland**	0.24-0.53	0.52	CM FN ID BK	CM FN ID BK
**Germany**	0.34-0.50	0.45	FN HC TC CC ID	FN HC TC CC ID
**Denmark**	0.34-0.56	0.40	OG FN CG UT HC ID	FN CG HC ID
**Spain**	0.49-0.50	0.45	CN FN EG CSR TC	CN FN EG CSR TC
**Finland**	0.33-0.48	0.43	FN ID CG TC BM	FN ID CG BM
**France**	0.31-0.57	0.46	BM CG CSR FN ID	BM CG CSR FN ID IT
**UK**	0.30-0.55	0.50	CSR BM FN ID OG	CSR FN ID
**Greece**	0.28-0.5	0.45	ID BR FN UT	ID BR FN UT OG
**Hong Kong**	0.31-0.52	0.46	CG FN ID PC BM	CG FN ID PC BM
**Indonesia**	0.33-0.57	0.57	ID CSR MF	CSR MF
**India**	0.25-0.53	0.42	AM BK PE CG1 MP OG PSU	BK PE CG1 MP OG PSU
**Japan**	0.39-0.55	0.43	EM RT TC SU TE TX WS	EM SU TE TX WS MP RB
**Sri Lanka**	0.33-0.54	0.45	FN MF	FN MF
**Malaysia**	0.38-0.55	0.45	CN ID PR TS IT	CP ID TS FN
**Netherlands**	0.32-0.57	0.52	BM FN ID	BM FN ID
**Norway**	0.32-0.44	0.43	FN EG ID BM	FN EG ID BM
**Philippines**	0.39-0.47	0.45	ID FN PR	ID FN PR
**Portugal**	0.30-0.48	0.45	CSR ID BM UT	CSR ID BM UT
**Qatar**	0.37-0.49	0.42	ID BF TP RE CG	ID BF RE CG
**Saudi Arabia**	0.23-0.49	0.44	BF BC PH CE ID MI RT	BF BC PH CE ID MI RT
**Sweden**	0.33-0.53	0.45	CG FN ID BM	CG FN ID BM
**Thailand**	0.34-0.48	0.45	ID PC BR CSR	ID PC BR CSR AF
**USA**	0.40-0.51	0.45	FN IT CS ID CD BM	FN IT CS ID CD BM
**South Africa**	0.31-0.48	0.45	FN CG CSR HC ID	CG CSR ID

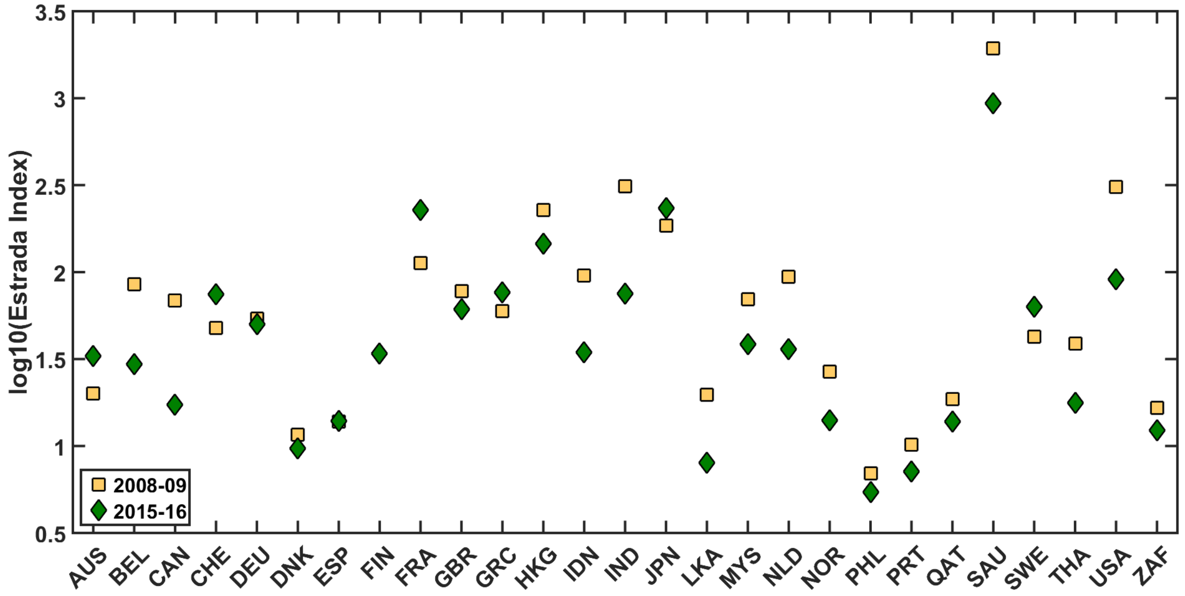

Figure 2. (Color online) Comparison of the normalized Estrada Indices for the years 2008-09 and 2015-16.

Table 2. Regression table: Dependent variable is the scaled eigenvector centrality and the independent variable is the scaled market capitalization (2015-16). *** : significant at 1%, **: at 5%, *: at 10%.

Countries	β_0	Tstat	Pvalue	β_1	Tstat	Pvalue	Rsquare
Australia	0.0000 (0.3481)	0.0000	0.9999	0.2131 (0.3692)	0.5773	0.5817	0.0454
Belgium	0.0000 (0.3695)	0.0000	0.9999	0.2517 (0.3951)	0.6371	0.5475	0.0633
Canada	0.0000 (0.3018)	0.0000	0.9999	0.0758 (0.3153)	0.2404	0.8148	0.0057
Denmark	0.0000 (0.2703)	0.0000	0.9999	0.5918 (0.2849)	2.0767	0.0714*	0.3502
Finland	0.0000 (0.2246)	0.0000	0.9999	0.7425 (0.2368)	3.1353	0.0139**	0.5513
France	0.0000 (0.2523)	0.0000	0.9999	0.7003 (0.2394)	2.7751	0.0241**	0.4904
Germany	0.0000 (0.2544)	0.0000	0.9999	0.6516 (0.2681)	2.4299	0.0412**	0.4246
Greece	0.0000 (0.1617)	0.0000	0.9999	-0.8759 (0.1705)	-5.1368	0.0008***	0.7673
Hong Kong	0.0000 (0.3042)	0.0000	0.9999	0.4208 (0.3207)	1.3123	0.2257	0.1771
India	0.0000 (0.3329)	0.0000	0.9999	0.1219 (0.3509)	0.3474	0.7373	0.0149
Indonesia	0.0000 (0.232)	0.0000	0.9999	0.7221 (0.2445)	2.9524	0.0183**	0.5214
Japan	0.0000 (0.3239)	0.0000	0.9999	0.2588 (0.3415)	0.7579	0.4702	0.0670
Malaysia	0.0000 (0.2719)	0.0000	0.9999	0.5853 (0.2866)	2.0421	0.0754*	0.3426
Netherlands	0.0000 (0.3292)	0.0000	0.9999	0.3821 (0.3492)	1.0941	0.3100	0.1460
Norway	0.0000 (0.2992)	0.0000	0.9999	0.4516 (0.3154)	1.4316	0.1901	0.2039
Philippines	0.0000 (0.1552)	0.0000	0.9999	0.9537 (0.1736)	5.4929	0.0118**	0.9095
Portugal	0.0000 (0.3133)	0.0000	0.9999	0.5715 (0.3349)	1.7061	0.1388	0.3266
Qatar	0.0000 (0.3742)	0.0000	0.9999	0.5723 (0.41)	1.3959	0.2352	0.3275
Saudi Arabia	0.0000 (0.2669)	0.0000	0.9999	0.2807 (0.277)	1.0133	0.3309	0.0788
South Africa	0.0000 (0.3739)	0.0000	0.9999	-0.2033 (0.3997)	-0.5086	0.6291	0.0413
Spain	0.0000 (0.2684)	0.0000	0.9999	0.1531 (0.5705)	0.8057	0.1577	0.0234
Sri Lanka	0.0000 (0.267)	0.0000	0.9999	0.5423 (0.28)	1.9363	0.0848*	0.2940
Sweden	0.0000 (0.2852)	0.0000	0.9999	0.5995 (0.3025)	1.9818	0.0879*	0.3594
Switzerland	0.0000 (0.2831)	0.0000	0.9999	0.4543 (0.2969)	1.5302	0.1603	0.2064
Thailand	0.0000 (0.3152)	0.0000	0.9999	0.5643 (0.337)	1.6743	0.1450	0.3184
UK	0.0000 (0.3132)	0.0000	0.9999	0.3575 (0.3301)	1.0830	0.3103	0.1278
USA	0.0000 (0.2728)	0.0000	0.9999	0.5817 (0.2875)	2.0228	0.0777*	0.3384

Table 3. Regression table: Dependent variable is the scaled eigenvector centrality and the independent variable is the scaled revenue (2015-16). *** : significant at 1%, **: at 5%, *: at 10%.

Countries	β_0	Tstat	Pvalue	β_1	Tstat	Pvalue	Rsquare
Australia	0.0000 (0.3144)	0.0000	0.9999	0.4704 (0.3335)	1.4106	0.2012	0.2213
Belgium	0.0000 (0.2247)	0.0000	0.9999	0.8084 (0.2402)	3.3644	0.0151**	0.6535
Canada	0.0000 (0.2916)	0.0000	0.9999	0.2689 (0.3045)	0.8830	0.3979	0.0723
Denmark	0.0000 (0.2837)	0.0000	0.9999	0.5333 (0.299)	1.7834	0.1123	0.2844
Finland	0.0000 (0.2988)	0.0000	0.9999	0.4542 (0.3149)	1.4422	0.1872	0.2063
France	0.0000 (0.236)	0.0000	0.9999	0.7104 (0.2487)	2.8557	0.0212**	0.5048
Germany	0.0000 (0.3203)	0.0000	0.9999	0.2964 (0.3376)	0.8778	0.4056	0.0878
Greece	0.0000 (0.3069)	0.0000	0.9999	-0.4029 (0.3235)	-1.2453	0.2482	0.1623
Hong Kong	0.0000 (0.2877)	0.0000	0.9999	0.5140 (0.3032)	1.6948	0.1285	0.2642
India	0.0000 (0.3158)	0.0000	0.9999	0.3368 (0.3329)	1.0117	0.3413	0.1134
Indonesia	0.0000 (0.233)	0.0000	0.9999	0.7193 (0.2456)	2.9286	0.0190**	0.5173
Japan	0.0000 (0.3137)	0.0000	0.9999	0.3531 (0.3307)	1.0678	0.3167	0.1247
Malaysia	0.0000 (0.2746)	0.0000	0.9999	0.5739 (0.2895)	1.9824	0.0827*	0.3294
Netherlands	0.0000 (0.3411)	0.0000	0.9999	0.2886 (0.3618)	0.7976	0.4512	0.0833
Norway	0.0000 (0.222)	0.0000	0.9999	0.7495 (0.234)	3.2031	0.0125**	0.5618
Philippines	0.0000 (0.4578)	0.0000	0.9999	0.4625 (0.5118)	0.9035	0.4328	0.2139
Portugal	0.0000 (0.2655)	0.0000	0.9999	0.7187 (0.2838)	2.5320	0.0445**	0.5165
Qatar	0.0000 (0.4536)	0.0000	0.9999	-0.1102 (0.4969)	-0.2219	0.8352	0.0121
Saudi Arabia	- -						
South Africa	0.0000 (0.3129)	0.0000	0.9999	0.5729 (0.3346)	1.7121	0.1377	0.3282
Spain	0.0000 (0.5099)	0.0000	0.9999	0.1577 (0.5701)	0.2767	0.7999	0.0248
Sri Lanka	0.0000 (0.243)	0.0000	0.9999	0.6442 (0.2549)	2.5267	0.0324**	0.4150
Sweden	0.0000 (0.2945)	0.0000	0.9999	0.5626 (0.3124)	1.8006	0.1147	0.3165
Switzerland	0.0000 (0.2646)	0.0000	0.9999	0.5537 (0.2775)	1.9952	0.0771*	0.3066
Thailand	0.0000 (0.3558)	0.0000	0.9999	0.3628 (0.3804)	0.9538	0.3770	0.1316
UK	0.0000 (0.3346)	0.0000	0.9999	-0.0688 (0.3527)	-0.1952	0.8500	0.0047
USA	0.0000 (0.2667)	0.0000	0.9999	0.6063 (0.2811)	2.1566	0.0631*	0.3676

Table 4. Regression table: Dependent variable is the scaled eigenvector centrality and the independent variable is the scaled number of employees (2015-16). *** : significant at 1%, **: at 5%, *: at 10%.

Countries	β_0	Tstat	Pvalue	β_1	Tstat	Pvalue	Rsquare
Australia	0.0000 (0.3038)	0.0000	0.9999	0.5225 (0.3222)	1.6213	0.1489	0.2730
Belgium	0.0000 (0.2631)	0.0000	0.9999	0.7247 (0.2812)	2.5766	0.0419**	0.5252
Canada	0.0000 (0.2919)	0.0000	0.9999	0.2652 (0.3049)	0.8698	0.4048	0.0703
Denmark	0.0000 (0.3164)	0.0000	0.9999	0.3316 (0.3335)	0.9942	0.3492	0.1099
Finland	0.0000 (0.2762)	0.0000	0.9999	0.5671 (0.2912)	1.9474	0.0873*	0.3216
France	0.0000 (0.1525)	0.0000	0.9999	0.8905 (0.1608)	5.5374	0.0005***	0.7930
Germany	0.0000 (0.2836)	0.0000	0.9999	0.5336 (0.299)	1.7845	0.1121	0.2847
Greece	0.0000 (0.1481)	0.0000	0.9999	-0.8971 (0.1561)	-5.7451	0.0004***	0.8049
Hong Kong	0.0000 (0.2471)	0.0000	0.9999	0.6759 (0.2605)	2.5943	0.0318**	0.4569
India	0.0000 (0.3354)	0.0000	0.9999	0.0189 (0.3535)	0.0534	0.9588	0.0003
Indonesia	0.0000 (0.2816)	0.0000	0.9999	0.5428 (0.2969)	1.8283	0.1049	0.2947
Japan	0.0000 (0.292)	0.0000	0.9999	0.4919 (0.3078)	1.5982	0.1486	0.2420
Malaysia	0.0000 (0.2975)	0.0000	0.9999	0.4615 (0.3136)	1.4717	0.1792	0.2130
Netherlands	0.0000 (0.2279)	0.0000	0.9999	0.7686 (0.2417)	3.1794	0.0155	0.5908
Norway	0.0000 (0.2736)	0.0000	0.9999	0.5784 (0.2884)	2.0054	0.0798*	0.3345
Philippines	0.0000 (0.4688)	0.0000	0.9999	0.4191 (0.5241)	0.7996	0.4823	0.1756
Portugal	0.0000 (0.3207)	0.0000	0.9999	0.5425 (0.3429)	1.5820	0.1647	0.2943
Qatar	- -						
Saudi Arabia	- -						
South Africa	0.0000 (0.3768)	0.0000	0.9999	0.1614 (0.4028)	0.4008	0.7024	0.0260
Spain	0.0000 (0.483)	0.0000	0.9999	0.3537 (0.5400)	0.6550	0.5591	0.1251
Sri Lanka	0.0000 (0.1924)	0.0000	0.9999	0.7958 (0.2018)	3.9435	0.0033***	0.6334
Sweden	0.0000 (0.315)	0.0000	0.9999	0.4673 (0.3341)	1.3986	0.2046	0.2184
Switzerland	0.0000 (0.2719)	0.0000	0.9999	0.5177 (0.2851)	1.8154	0.1028	0.2680
Thailand	- -						
UK	0.0000 (0.255)	0.0000	0.9999	0.6495 (0.2688)	2.4165	0.0420**	0.4219
USA	0.0000 (0.2529)	0.0000	0.9999	0.6566 (0.2666)	2.4628	0.0391**	0.4312

Table 5. Regression table: Dependent variable is the scaled eigenvector centrality and the independent variable is the scaled market capitalization (2012-13). *** : significant at 1%, **: at 5%, *: at 10%.

Countries	β_0	Tstat	Pvalue	β_1	Tstat	Pvalue	Rsquare
Australia	0.0000 (0.3297)	0.0000	0.9999	0.3792 (0.3497)	1.0841	0.3142	0.1438
Belgium	0.0000 (0.3696)	0.0000	0.9999	0.2511 (0.3952)	0.6355	0.5486	0.0631
Canada	0.0000 (0.2765)	0.0000	0.9999	0.4076 (0.2888)	1.4116	0.1884	0.1661
Denmark	0.0000 (0.2484)	0.0000	0.9999	0.6718 (0.2619)	2.5652	0.0334**	0.4513
Finland	0.0000 (0.2854)	0.0000	0.9999	0.5252 (0.3009)	1.7458	0.1190	0.2759
France	0.0000 (0.2788)	0.0000	0.9999	0.5559 (0.2939)	1.8914	0.0952*	0.3090
Germany	0.0000 (0.2906)	0.0000	0.9999	0.4995 (0.3063)	1.6309	0.1415	0.2495
Greece	0.0000 (0.2915)	0.0000	0.9999	-0.4947 (0.3073)	-1.6098	0.1461	0.2447
Hong Kong	0.0000 (0.3004)	0.0000	0.9999	0.4451 (0.3166	1.4059	0.1974	0.1981
India	0.0000 (0.3268)	0.0000	0.9999	-0.2246 (0.3445)	-0.6521	0.5327	0.0505
Indonesia	0.0000 (0.2521)	0.0000	0.9999	0.6598 (0.2657)	2.4833	0.0379**	0.4353
Japan	0.0000 (0.3225)	0.0000	0.9999	0.2753 (0.3399	0.8099	0.4414	0.0758
Malaysia	0.0000 (0.2447)	0.0000	0.9999	0.6839 (0.2579)	2.6513	0.0292**	0.4677
Netherlands	0.0000 (0.3396)	0.0000	0.9999	0.3031 (0.3602)	0.8415	0.4279	0.0919
Norway	0.0000 (0.2874)	0.0000	0.9999	0.5154 (0.3030	1.7013	0.1273	0.2657
Philippines	0.0000 (0.2813)	0.0000	0.9999	0.8386 (0.3145)	2.6664	0.0759*	0.7033
Portugal	0.0000 (0.3219)	0.0000	0.9999	0.5379 (0.3442)	1.5630	0.1691	0.2893
Qatar	0.0000 (0.3444)	0.0000	0.9999	0.6561 (0.3773)	1.7389	0.1570	0.4305
Saudi Arabia	0.0000 (0.2605)	0.0000	0.9999	0.3506 (0.2703)	1.2970	0.2190	0.1229
South Africa	0.0000 (0.3812)	0.0000	0.9999	0.0589 (0.4075)	0.1446	0.8897	0.0035
Spain	0.0000 (0.3491	0.0000	0.9999	0.7369 (0.3903)	1.8882	0.1554	0.5430
Sri Lanka	0.0000 (0.3142)	0.0000	0.9999	0.1512 (0.3295)	0.4589	0.6572	0.0229
Sweden	0.0000 (0.3320)	0.0000	0.9999	0.3630 (0.3522)	1.0309	0.3369	0.1318
Switzerland	0.0000 (0.3037)	0.0000	0.9999	0.2947 (0.3185)	0.9250	0.3791	0.0868
Thailand	0.0000 (0.3076)	0.0000	0.9999	0.5925 (0.3289)	1.8018	0.1217	0.3511
UK	0.0000 (0.2792)	0.0000	0.9999	0.5540 (0.2943)	1.8821	0.0966*	0.3069
USA	0.0000 (0.2798)	0.0000	0.9999	0.5516 (0.2949)	1.8704	0.0984*	0.3042

Table 6. Regression table: Dependent variable is the scaled eigenvector centrality and the independent variable is the scaled revenue (2012-13). *** : significant at 1%, **: at 5%, *: at 10%.

Countries	β_0	Tstat	Pvalue	β_1	Tstat	Pvalue	Rsquare
Australia	0.0000<br>(0.3128)	0.0000	0.9999	0.4789<br>(0.3318)	1.4434	0.1921	0.2294
Belgium	0.0000<br>(0.3473)	0.0000	0.9999	0.4160<br>(0.3713)	1.1205	0.3053	0.1730
Canada	0.0000<br>(0.2706)	0.0000	0.9999	0.4482<br>(0.2827)	1.5857	0.1439	0.2009
Denmark	0.0000<br>(0.2630)	0.0000	0.9999	0.6207<br>(0.2772)	2.2391	0.0555*	0.3853
Finland	0.0000<br>(0.3067)	0.0000	0.9999	0.4050<br>(0.3233)	1.2528	0.2457	0.1640
France	0.0000<br>(0.2533)	0.0000	0.9999	0.6554<br>(0.2670)	2.4546	0.0397**	0.4296
Germany	0.0000<br>(0.3284)	0.0000	0.9999	0.2033<br>(0.3462)	0.5873	0.5732	0.0413
Greece	0.0000<br>(0.3132)	0.0000	0.9999	-0.3581<br>(0.3301)	-1.0848	0.3096	0.1282
Hong Kong	0.0000<br>(0.2759)	0.0000	0.9999	0.5687<br>(0.2908)	1.9588	0.0862*	0.3235
India	0.0000<br>(0.3246)	0.0000	0.9999	0.2516<br>(0.3422)	0.7352	0.4832	0.0633
Indonesia	0.0000<br>(0.2361)	0.0000	0.9999	0.7102<br>(0.2489)	2.8534	0.0214**	0.5044
Japan	0.0000<br>(0.3124)	0.0000	0.9999	0.3641<br>(0.3293)	1.1056	0.3010	0.1325
Malaysia	0.0000<br>(0.2471)	0.0000	0.9999	0.6761<br>(0.2605)	2.5955	0.0318**	0.4571
Netherlands	0.0000<br>(0.3505)	0.0000	0.9999	0.1811<br>(0.3717)	0.4871	0.6410	0.0328
Norway	0.0000<br>(0.2282)	0.0000	0.9999	0.7328<br>(0.2406	3.0461	0.0159**	0.5370
Philippines	0.0000<br>(0.4494)	0.0000	0.9999	0.4925<br>(0.5025)	0.9801	0.3993	0.2425
Portugal	0.0000<br>(0.3433)	0.0000	0.9999	0.4379<br>(0.3670)	1.1931	0.2778	0.1918
Qatar	0.0000<br>(0.4416)	0.0000	0.9999	-0.2533<br>(0.4837)	-0.5237	0.6282	0.0642
Saudi Arabia	0.0000<br>(0.2654)	0.0000	0.9999	0.2991<br>(0.2755)	1.0860	0.2988	0.0895
South Africa	0.0000<br>(0.3336)	0.0000	0.9999	0.4865<br>(0.3567)	1.3639	0.2216	0.2367
Spain	0.0000<br>(0.4486)	0.0000	0.9999	0.4954<br>(0.5015)	0.9877	0.3961	0.2454
Sri Lanka	0.0000<br>(0.2864)	0.0000	0.9999	0.4335<br>(0.3004)	1.4432	0.1829	0.1879
Sweden	0.0000<br>(0.3252)	0.0000	0.9999	0.4090<br>(0.3449	1.1857	0.2744	0.1673
Switzerland	0.0000<br>(0.2861)	0.0000	0.9999	0.4353<br>(0.3001	1.4504	0.1809	0.1895
Thailand	0.0000<br>(0.3777)	0.0000	0.9999	0.1469<br>(0.4038)	0.3637	0.7286	0.0216
UK	0.0000<br>(0.3153)	0.0000	0.9999	0.3413<br>(0.3323)	1.0270	0.3345	0.1165
USA	0.0000<br>(0.2412)	0.0000	0.9999	0.6949<br>(0.2542)	2.7331	0.0257**	0.4829

Table 7. Regression table: Dependent variable is the scaled eigenvector centrality and the independent variable is the scaled employees (2012-13). *** : significant at 1%, **: at 5%, *: at 10%.

Countries	β_0	Tstat	Pvalue	β_1	Tstat	Pvalue	Rsquare
Australia	0.0000 (0.3108)	0.0000	0.9999	0.4890 (0.3297)	1.4833	0.1816	0.2391
Belgium	0.0000 (0.3619)	0.0000	0.9999	0.3196 (0.3868)	0.8262	0.4403	0.1021
Canada	0.0000 (0.2901)	0.0000	0.9999	0.2865 (0.3030)	0.9458	0.3665	0.0821
Denmark	0.0000 (0.2930)	0.0000	0.9999	0.4865 (0.3089)	1.5749	0.1539	0.2367
Finland	0.0000 (0.2804)	0.0000	0.9999	0.5489 (0.2955)	1.8574	0.1003	0.3013
France	0.0000 (0.2689)	0.0000	0.9999	0.5977 (0.2834)	2.1087	0.0680*	0.3573
Germany	0.0000 (0.3068)	0.0000	0.9999	0.4042 (0.3234)	1.2500	0.2466	0.1634
Greece	0.0000 (0.2968)	0.0000	0.9999	-0.4656 (0.3129)	-1.4881	0.1750	0.2168
Hong Kong	0.0000 (0.2623)	0.0000	0.9999	0.6232 (0.2765)	2.2539	0.0542*	0.3884
India	0.0000 (0.3352)	0.0000	0.9999	-0.0344 (0.3533)	-0.0975	0.9247	0.0012
Indonesia	0.0000 (0.2743)	0.0000	0.9999	0.5755 (0.2891)	1.9905	0.0817*	0.3312
Japan	0.0000 (0.2857)	0.0000	0.9999	0.5239 (0.3011)	1.7398	0.1201	0.2745
Malaysia	0.0000 (0.2831)	0.0000	0.9999	0.5362 (0.2984)	1.7965	0.1101	0.2875
Netherlands	0.0000 (0.2626)	0.0000	0.9999	0.6760 (0.2785)	2.4273	0.0456**	0.4570
Norway	0.0000 (0.2334)	0.0000	0.9999	0.7181 (0.2461)	2.9182	0.0193**	0.5156
Philippines	0.0000 (0.4480	0.0000	0.9999	0.4974 (0.5008)	0.9932	0.3938	0.2475
Portugal	0.0000 (0.3730)	0.0000	0.9999	0.2149 (0.3987)	0.5391	0.6092	0.0462
Qatar	- -						
Saudi Arabia	- -						
South Africa	0.0000 (0.3632)	0.0000	0.9999	0.3089 (0.3883)	0.7955	0.4566	0.0954
Spain	0.0000 (0.5162)	0.0000	0.9999	0.0259 (0.5772)	0.0449	0.9670	0.0000
Sri Lanka	0.0000 (0.2675)	0.0000	0.9999	0.5402 (0.2805)	1.9257	0.0863*	0.2918
Sweden	0.0000 (0.3342)	0.0000	0.9999	0.3467 (0.3545)	0.9780	0.3606	0.1202
Switzerland	0.0000 (0.2882)	0.0000	0.9999	0.4218 (0.3022)	1.3956	0.1963	0.1779
Thailand	0.0000 (0.2881)	0.0000	0.9999	0.6564 (0.3080)	2.1315	0.0771*	0.4309
UK	0.0000 (0.2084)	0.0000	0.9999	0.7835 (0.2197)	3.5663	0.0073	0.6139
USA	0.0000 (0.2782)	0.0000	0.9999	0.5586 (0.2933)	1.9047	0.0933*	0.3120

Table 8. Regression table: Dependent variable is the scaled eigenvector centrality and the independent variable is the scaled market capitalization (2008-09). *** : significant at 1%, **: at 5%, *: at 10%.

Countries	β_0	Tstat	Pvalue	β_1	Tstat	Pvalue	Rsquare
Australia	0.0000 (0.3507)	0.0000	0.9999	0.1770 (0.3719)	0.4759	0.6485	0.0313
Belgium	0.0000 (0.3791)	0.0000	0.9999	-0.1193 (0.4053)	-0.2943	0.7783	0.0142
Canada	0.0000 (0.3027)	0.0000	0.9999	0.0000 (0.3162)	0.0001	0.9998	0.0000
Denmark	0.0000 (0.3058)	0.0000	0.9999	0.4103 (0.3224)	1.2727	0.2388	0.1683
Finland	- -						
France	0.0000 (0.3074)	0.0000	0.9999	0.3995 (0.3241)	1.2329	0.2526	0.1596
Germany	0.0000 (0.2716)	0.0000	0.9999	0.5865 (0.2863)	2.0484	0.0746*	0.3440
Greece	0.0000 (0.3327)	0.0000	0.9999	0.1258 (0.3507)	0.3587	0.7290	0.0158
Hong Kong	0.0000 (0.3173)	0.0000	0.9999	0.3239 (0.3344)	0.9684	0.3611	0.1049
India	0.0000 (0.2756)	0.0000	0.9999	0.5696 (0.2905)	1.9605	0.0855*	0.3245
Indonesia	0.0000 (0.2925)	0.0000	0.9999	0.4891 (0.3083)	1.5861	0.1513	0.2392
Japan	0.0000 (0.334)	0.0000	0.9999	0.0915 (0.3520)	0.2600	0.8013	0.0083
Malaysia	0.0000 (0.273)	0.0000	0.9999	0.5809 (0.2877)	2.0188	0.0782*	0.3375
Netherlands	0.0000 (0.3165)	0.0000	0.9999	0.4594 (0.3357)	1.3686	0.2134	0.2110
Norway	0.0000 (0.3016)	0.0000	0.9999	0.4369 (0.318)	1.3739	0.2067	0.1909
Philippines	0.0000 (0.4176)	0.0000	0.9999	0.5880 (0.4669)	1.2592	0.2970	0.3457
Portugal	0.0000 (0.3116)	0.0000	0.9999	0.5779 (0.3331)	1.7346	0.1334	0.3340
Qatar	0.0000 (0.3914)	0.0000	0.9999	0.5140 (0.4288)	1.1987	0.2967	0.2642
Saudi Arabia	0.0000 (0.2714)	0.0000	0.9999	0.2179 (0.2817)	0.7734	0.4542	0.0474
South Africa	0.0000 (0.3802)	0.0000	0.9999	-0.0932 (0.4064)	-0.2293	0.8262	0.0086
Spain	0.0000 (0.4885)	0.0000	0.9999	0.3238 (0.5462)	0.5928	0.5950	0.1048
Sri Lanka	0.0000 (0.301)	0.0000	0.9999	-0.2309 (0.3243)	-0.7120	0.4945	0.0533
Sweden	0.0000 (0.3435)	0.0000	0.9999	0.3643 (0.2658)	0.7297	0.4892	0.0706
Switzerland	0.0000 (0.3173)	0.0000	0.9999	-0.0532 (0.3328)	-0.1599	0.8764	0.0028
Thailand	0.0000 (0.3677)	0.0000	0.9999	0.2691 (0.3931)	0.6845	0.5191	0.0724
UK	0.0000 (0.3346)	0.0000	0.9999	0.0691 (0.3527)	0.1959	0.8495	0.0047
USA	0.0000 (0.3315)	0.0000	0.9999	0.1508 (0.3495)	0.4315	0.6774	0.0227

Table 9. Regression table: Dependent variable is the scaled eigenvector centrality and the independent variable is the scaled revenue (2008-09). *** : significant at 1%, **: at 5%, *: at 10%.

Countries	β_0	Tstat	Pvalue	β_1	Tstat	Pvalue	Rsquare
Australia	0.0000 (0.335)	0.0000	0.9999	0.3408 (0.3553)	0.9592	0.3694	0.1161
Belgium	0.0000 (0.3676)	0.0000	0.9999	-0.2708 (0.3929)	-0.6891	0.5164	0.0733
Canada	0.0000 (0.2869)	0.0000	0.9999	0.3194 (0.2996)	1.0658	0.3115	0.1020
Denmark	0.0000 (0.2908)	0.0000	0.9999	0.4982 (0.3065)	1.6254	0.1427	0.2482
Finland	- -						
France	0.0000 (0.2457)	0.0000	0.9999	0.6805 (0.259)	2.6273	0.0303**	0.4631
Germany	0.0000 (0.2792)	0.0000	0.9999	0.5538 (0.2943)	1.8814	0.0966*	0.3067
Greece	0.0000 (0.3071)	0.0000	0.9999	-0.4015 (0.3237)	-1.2401	0.2500	0.1612
Hong Kong	0.0000 (0.2956)	0.0000	0.9999	0.4719 (0.3116)	1.5142	0.1684	0.2227
India	0.0000 (0.3022)	0.0000	0.9999	0.4333 (0.3186)	1.3601	0.2108	0.1878
Indonesia	0.0000 (0.2747)	0.0000	0.9999	0.5734 (0.2896)	1.9798	0.0830*	0.3288
Japan	0.0000 (0.3226)	0.0000	0.9999	0.2727 (0.3401)	0.8018	0.4458	0.0743
Malaysia	0.0000 (0.2758)	0.0000	0.9999	0.5687 (0.2908)	1.9555	0.0862*	0.3234
Netherlands	0.0000 (0.3406)	0.0000	0.9999	0.2934 (0.3613)	0.8122	0.4433	0.0861
Norway	0.0000 (0.2482)	0.0000	0.9999	0.6723 (0.2617)	2.5688	0.0331**	0.4520
Philippines	0.0000 (0.4791)	0.0000	0.9999	0.3728 (0.5357)	0.6959	0.5365	0.1389
Portugal	0.0000 (0.2975)	0.0000	0.9999	0.6268 (0.318)	1.9705	0.0962*	0.3928
Qatar	0.0000 (0.3915)	0.0000	0.9999	-0.5138 (0.4289)	-1.1979	0.2970	0.2640
Saudi Arabia	0.0000 (0.274)	0.0000	0.9999	0.1724 (0.2843)	0.6064	0.5555	0.0297
South Africa	0.0000 (0.3191)	0.0000	0.9999	0.5492 (0.3411)	1.6100	0.1585	0.3017
Spain	0.0000 (0.367)	0.0000	0.9999	0.7033 (0.4103)	1.7139	0.1850	0.4947
Sri Lanka	0.0000 (0.3167)	0.0000	0.9999	-0.0857 (0.3321)	-0.2579	0.8022	0.0073
Sweden	0.0000 (0.3071)	0.0000	0.9999	0.5071 (0.3257)	1.5566	0.1634	0.2571
Switzerland	0.0000 (0.3153)	0.0000	0.9999	0.1245 (0.3307)	0.3765	0.7152	0.0155
Thailand	0.0000 (0.3748)	0.0000	0.9999	0.1908 (0.4007)	0.4761	0.6507	0.0364
UK	0.0000 (0.3293)	0.0000	0.9999	0.1884 (0.3472)	0.5427	0.6020	0.0355
USA	0.0000 (0.3319)	0.0000	0.9999	0.1431 (0.3499)	0.4091	0.6931	0.0205

Table 10. Regression table: Dependent variable is the scaled eigenvector centrality and the independent variable is the scaled number of employees (2008-09). *** : significant at 1%, **: at 5%, *: at 10%.

Countries	β_0	Tstat	Pvalue	β_1	Tstat	Pvalue	Rsquare
Australia	0.0000 (0.3369)	0.0000	0.9999	0.3251 (0.3574)	0.9095	0.3932	0.1056
Belgium	0.0000 (0.3802)	0.0000	0.9999	-0.0918 (0.4065)	-0.2259	0.8287	0.0084
Canada	0.0000 (0.2953)	0.0000	0.9999	0.2206 (0.3084)	0.7154	0.4907	0.0486
Denmark	0.0000 (0.3154)	0.0000	0.9999	0.3400 (0.3324)	1.0226	0.3364	0.1156
Finland	- -						
France	0.0000 (0.2558)	0.0000	0.9999	0.6465 (0.2697)	2.3971	0.0433**	0.4180
Germany	0.0000 (0.2916)	0.0000	0.9999	0.4939 (0.3074)	1.6066	0.1468	0.2439
Greece	0.0000 (0.3353)	0.0000	0.9999	-0.0177 (0.3534)	-0.0502	0.9611	0.0003
Hong Kong	0.0000 (0.2864)	0.0000	0.9999	0.5200 (0.3019)	1.7222	0.1233	0.2704
India	0.0000 (0.3088)	0.0000	0.9999	0.3903 (0.3255)	1.1991	0.2647	0.1523
Indonesia	0.0000 (0.2749)	0.0000	0.9999	0.5726 (0.2898)	1.9759	0.0835*	0.3279
Japan	0.0000 (0.2938)	0.0000	0.9999	0.4823 (0.3096)	1.5575	0.1579	0.2326
Malaysia	0.0000 (0.2835)	0.0000	0.9999	0.5341 (0.2988)	1.7870	0.1117	0.2853
Netherlands	0.0000 (0.3165)	0.0000	0.9999	0.4592 (0.3357)	1.3679	0.2136	0.2109
Norway	0.0000 (0.2467)	0.0000	0.9999	0.6772 (0.2601)	2.6034	0.0314**	0.4586
Philippines	0.0000 (0.4808)	0.0000	0.9999	0.3644 (0.5376)	0.6777	0.5465	0.1327
Portugal	0.0000 (0.329)	0.0000	0.9999	0.5074 (0.3517)	1.4425	0.1992	0.2575
Qatar	- -						
Saudi Arabia	- -						
South Africa	0.0000 (0.364)	0.0000	0.9999	0.3017 (0.3892)	0.7753	0.4675	0.0910
Spain	0.0000 (0.4049)	0.0000	0.9999	0.6205 (0.4527)	1.3707	0.2640	0.3851
Sri Lanka	0.0000 (0.2997)	0.0000	0.9999	0.3330 (0.3143)	1.0594	0.3170	0.1109
Sweden	0.0000 (0.3213)	0.0000	0.9999	0.4321 (0.3408)	1.2679	0.2453	0.1867
Switzerland	0.0000 (0.3083)	0.0000	0.9999	0.2419 (0.3234)	0.7481	0.4734	0.0585
Thailand	0.0000 (0.3618)	0.0000	0.9999	-0.3195 (0.3868)	-0.8259	0.4404	0.1020
UK	0.0000 (0.2444)	0.0000	0.9999	0.6847 (0.2576)	2.6571	0.0289**	0.4688
USA	0.0000 (0.2776)	0.0000	0.9999	0.5610 (0.2926)	1.9171	0.0915*	0.3148

Table 11. Regression table: Dependent variable is the scaled subgraph centrality and the independent variable is the scaled number of market capitalization (2015-16). *** : significant at 1%, **: at 5%, *: at 10%.

Countries	β_0	Tstat	Pvalue	β_1	Tstat	Pvalue	Rsquare
Australia	0 (0.3471)	0	1	0.2264 (0.3682)	0.6149	0.5581	0.0513
Belgium	0 (0.3709)	0	1	0.2383 (0.3965)	0.601	0.5699	0.0568
Canada	0 (0.2998)	0	1	0.1415 (0.3131)	0.452	0.661	0.0201
Switzerland	0 (0.2819)	0	1	0.4624 (0.2956)	1.5644	0.1522	0.2138
Germany	0 (0.2314)	0	1	0.724 (0.2439)	2.9686	0.018**	0.5242
Denmark	0 (0.247)	0	1	0.6766 (0.2604)	2.5989	0.0317**	0.4578
Spain	0 (0.5107)	0	1	0.1492 (0.5709)	0.2613	0.8109	0.0223
Finland	0 (0.2177)	0	1	0.761 (0.2294)	3.3176	0.0106**	0.5791
France	0 (0.2377)	0	1	0.7057 (0.2506)	2.8169	0.0227	0.498
UK	0 (0.3125)	0	1	0.3638 (0.3294)	1.1046	0.3015	0.1324
Greece	0 (0.1649)	0	1	-0.871 (0.1738)	-5.0127	0.0011***	0.7586
Hong Kong	0 (0.3032)	0	1	0.4281 (0.3196)	1.3396	0.2172	0.1833
Indonesia	0 (0.2235)	0	1	0.7457 (0.2356)	3.1651	0.0133**	0.556
India	0 (0.3305)	0	1	0.1706 (0.3484)	0.4896	0.6376	0.0291
Japan	0 (0.3238)	0	1	0.2616 (0.3413)	0.7665	0.4655	0.0685
Sri Lanka	0 (0.2587)	0	1	0.5812 (0.2713)	2.1426	0.0608*	0.3378
Malaysia	0 (0.2483)	0	1	0.6724 (0.2618)	2.5688	0.0332**	0.452
Netherlands	0 (0.3328)	0	1	0.3582 (0.3529)	1.0148	0.344	0.1283
Norway	0 (0.3029)	0	1	0.4299 (0.3193)	1.3467	0.215	0.1849
Philippines	0 (0.1622)	0	1	0.9495 (0.1813)	5.237	0.0136**	0.9015
Portugal	0 (0.3144)	0	1	0.5679 (0.3361)	1.6898	0.1421	0.3225
Qatar	0 (0.3746)	0	1	0.5716 (0.4103)	1.3932	0.2361	0.3268
Saudi Arabia	0 (0.2672)	0	1	0.2783 (0.2773)	1.0035	0.3355	0.0775
Sweden	0 (0.2769)	0	1	0.6297 (0.2937)	2.1445	0.0692*	0.3965
Thailand	0 (0.3157)	0	1	0.5628 (0.3375)	1.6674	0.1465	0.3167
USA	0 (0.2705)	0	1	0.5914 (0.2852)	2.0741	0.0718*	0.3497
South Africa	0 (0.3735)	0	1	-0.2091 (0.3993)	-0.5237	0.6194	0.0437

Table 12. Regression table: Dependent variable is the scaled subgraph centrality and the independent variable is the scaled revenue (2015-16). *** : significant at 1%, **: at 5%, *: at 10%.

Countries	β_0	Tstat	Pvalue	β_1	Tstat	Pvalue	Rsquare
Australia	0 (0.3108)	0	1	0.4896 (0.3296)	1.4856	0.181	0.2398
Belgium	0 (0.2269)	0	1	0.8045 (0.2426)	3.3174	0.0161**	0.6472
Canada	0 (0.2875)	0	1	0.3143 (0.3003)	1.0468	0.3199	0.0988
Switzerland	0 (0.2584)	0	1	0.5824 (0.271)	2.1489	0.0602*	0.3391
Germany	0 (0.3193)	0	1	0.3065 (0.3366)	0.9106	0.3892	0.094
Denmark	0 (0.2714)	0	1	0.5879 (0.2861)	2.0553	0.074*	0.3456
Spain	0 (0.5103)	0	1	0.1535 (0.5706)	0.2691	0.8054	0.0236
Finland	0 (0.2967)	0	1	0.467 (0.3127)	1.4935	0.1737	0.2181
France	0 (0.2342)	0	1	0.716 (0.2469)	2.901	0.0199**	0.5127
UK	0 (0.335)	0	1	-0.0501 (0.3532)	-0.1417	0.8909	0.0026
Greece	0 (0.3103)	0	1	-0.3799 (0.3271)	-1.1613	0.2791	0.1443
Hong Kong	0 (0.2868)	0	1	0.519 (0.3023)	1.7172	0.1243	0.2694
Indonesia	0 (0.2239)	0	1	0.7449 (0.236)	3.1571	0.0135**	0.5548
India	0 (0.3123)	0	1	0.3648 (0.3292)	1.1082	0.3001	0.1331
Japan	0 (0.3121)	0	1	0.3669 (0.3289)	1.1156	0.2971	0.1347
Sri Lanka	0 (0.2321)	0	1	0.6833 (0.2434)	2.8073	0.0205**	0.4669
Malaysia	0 (0.2506)	0	1	0.665 (0.2641)	2.5182	0.036**	0.4422
Netherlands	0 (0.3423)	0	1	0.2787 (0.363)	0.7677	0.4678	0.0777
Norway	0 (0.2196)	0	1	0.7561 (0.2315)	3.2671	0.0115**	0.5716
Philippines	0 (0.4627)	0	1	0.4441 (0.5173)	0.8585	0.4538	0.1973
Portugal	0 (0.2637)	0	1	0.7236 (0.2819)	2.5675	0.0425**	0.5236
Qatar	0 (0.454)	0	1	-0.1043 (0.4973)	-0.2096	0.8443	0.0109
Saudi Arabia	- -						
Sweden	0 (0.2877)	0	1	0.5905 (0.3051)	1.9356	0.0942*	0.3487
Thailand	0 (0.3586)	0	1	0.3443 (0.3833)	0.8983	0.4037	0.1186
USA	0 (0.2679)	0	1	0.602 (0.2824)	2.1321	0.0656*	0.3624
South Africa	0 (0.3154)	0	1	0.5642 (0.3371)	1.6737	0.1453	0.3183

Table 13. Regression table: Dependent variable is the scaled subgraph centrality and the independent variable is the scaled number of employees (2015-16). *** : significant at 1%, **: at 5%, *: at 10%.

Countries	β_0	Tstat	Pvalue	β_1	Tstat	Pvalue	Rsquare
Australia	0 (0.3004)	0	1	0.5382 (0.3186)	1.6895	0.135	0.2897
Belgium	0 (0.2639)	0	1	0.7229 (0.2822)	2.5624	0.0428**	0.5226
Canada	0 (0.2892)	0	1	0.2968 (0.302)	0.9829	0.3489	0.0881
Switzerland	0 (0.2642)	0	1	0.5561 (0.2771)	2.0069	0.0758*	0.3092
Germany	0 (0.2682)	0	1	0.6009 (0.2827)	2.1258	0.0663*	0.361
Denmark	0 (0.3127)	0	1	0.362 (0.3296)	1.0983	0.3041	0.1311
Spain	0 (0.4829)	0	1	0.3547 (0.5399)	0.6571	0.5581	0.1258
Finland	0 (0.2737)	0	1	0.5782 (0.2885)	2.0044	0.08*	0.3344
France	0 (0.1474)	0	1	0.8983 (0.1554)	5.7815	0.0005***	0.8069
UK	0 (0.2467)	0	1	0.6776 (0.2601)	2.6056	0.0314**	0.4591
Greece	0 (0.1679)	0	1	-0.8658 (0.177)	-4.892	0.0013***	0.7495
Hong Kong	0 (0.2432)	0	1	0.6887 (0.2564)	2.6866	0.0277**	0.4743
Indonesia	0 (0.2734)	0	1	0.5797 (0.2882)	2.0118	0.0791*	0.336
India	0 (0.3347)	0	1	0.0685 (0.3528)	0.1941	0.851	0.0047
Japan	0 (0.2892)	0	1	0.5066 (0.3049)	1.6619	0.1352	0.2567
Sri Lanka	0 (0.1655)	0	1	0.8539 (0.1736)	4.9213	0.0009***	0.7291
Malaysia	0 (0.2802)	0	1	0.5499 (0.2954)	1.862	0.0997*	0.3024
Netherlands	0 (0.2201)	0	1	0.7867 (0.2334)	3.3712	0.012**	0.6189
Norway	0 (0.2771)	0	1	0.5636 (0.2921)	1.9298	0.0898*	0.3177
Philippines	0 (0.4776)	0	1	0.3804 (0.534)	0.7123	0.5278	0.1447
Portugal	0 (0.3201)	0	1	0.5457 (0.3422)	1.595	0.1619	0.2978
Qatar	- -						
Saudi Arabia	- -						
Sweden	0 (0.3099)	0	1	0.4941 (0.3287)	1.5035	0.1765	0.2441
Thailand	- -						
USA	0 (0.248)	0	1	0.6735 (0.2614)	2.5767	0.0328**	0.4536
South Africa	0 (0.3771)	0	1	0.1591 (0.4031)	0.3947	0.7068	0.0253

Table 14. Regression table: Dependent variable is the scaled subgraph centrality and the independent variable is the scaled market capitalization (2012-13). *** : significant at 1%, **: at 5%, *: at 10%.

Countries	β_0	Tstat	Pvalue	β_1	Tstat	Pvalue	Rsquare
Australia	0.0000 (0.3309)	0.0000	0.9999	0.3713 (0.3509)	1.0581	0.3251	0.1379
Belgium	0.0000 (0.3696)	0.0000	0.9999	0.2513 (0.3951)	0.6360	0.5483	0.0632
Canada	0.0000 (0.2704)	0.0000	0.9999	0.4497 (0.2825)	1.5920	0.1425	0.2022
Denmark	0.0000 (0.2288	0.0000	0.9999	0.7311 (0.2412)	3.0311	0.0163**	0.5345
Finland	0.0000 (0.2774)	0.0000	0.9999	0.5621 (0.2924)	1.9223	0.0908	0.3160
France	0.0000 (0.2790)	0.0000	0.9999	0.5550 (0.2941)	1.8870	0.0959*	0.3080
Germany	0.0000 (0.2772)	0.0000	0.9999	0.5629 (0.2922)	1.9265	0.0902*	0.3169
Greece	0.0000 (0.2942)	0.0000	0.9999	-0.4801 (0.3101)	-1.5482	0.1602	0.2305
Hong Kong	0.0000 (0.2961)	0.0000	0.9999	0.4698 (0.3121	1.5053	0.1707	0.2207
India	0.0000 (0.3312)	0.0000	0.9999	-0.1571 (0.3492	-0.4498	0.6648	0.0247
Indonesia	0.0000 (0.2439)	0.0000	0.9999	0.6864 (0.2571)	2.6697	0.0284**	0.4712
Japan	0.0000 (0.3219)	0.0000	0.9999	0.2810 (0.3393)	0.8282	0.4316	0.0790
Malaysia	0.0000 (0.2262)	0.0000	0.9999	0.7385 (0.2384)	3.0979	0.0147**	0.5454
Netherlands	0.0000 (0.3465)	0.0000	0.9999	0.2332 (0.3675)	0.6344	0.5460	0.0544
Norway	0.0000 (0.2721)	0.0000	0.9999	0.5846 (0.2868)	2.0379	0.0759*	0.3417
Philippines	0.0000 (0.2874)	0.0000	0.9999	0.8308 (0.3214)	2.5852	0.0814*	0.6902
Portugal	0.0000 (0.3276)	0.0000	0.9999	0.5139 (0.3502)	1.4673	0.1927	0.2641
Qatar	0.0000 (0.3348)	0.0000	0.9999	0.6796 (0.3668)	1.8528	0.1375	0.4618
Saudi Arabia	0.0000 (0.2608)	0.0000	0.9999	0.3479 (0.2706)	1.2854	0.2229	0.1210
South Africa	0.0000 (0.3818)	0.0000	0.9999	-0.0246 (0.4081)	-0.0604	0.9538	0.0006
Spain	0.0000 (0.3468	0.0000	0.9999	0.7409 (0.3877)	1.9109	0.1520	0.5490
Sri Lanka	0.0000 (0.3108)	0.0000	0.9999	0.2088 (0.3260)	0.6406	0.5377	0.0436
Sweden	0.0000 (0.3269)	0.0000	0.9999	0.3979 (0.3468)	1.1475	0.2889	0.1583
Switzerland	0.0000 (0.3074)	0.0000	0.9999	0.2539 (0.3224)	0.7876	0.4512	0.0645
Thailand	0.0000 (0.3104)	0.0000	0.9999	0.5825 (0.3318)	1.7553	0.1297	0.3393
UK	0.0000 (0.2792)	0.0000	0.9999	0.5543 (0.2943)	1.8835	0.0964*	0.3072
USA	0.0000 (0.2830)	0.0000	0.9999	0.5367 (0.2983)	1.7992	0.1097	0.2881

Table 15. Regression table: Dependent variable is the scaled subgraph centrality and the independent variable is the scaled revenue (2012-13). *** : significant at 1%, **: at 5%, *: at 10%.

Countries	β_0	Tstat	Pvalue	β_1	Tstat	Pvalue	Rsquare
Australia	0.0000 (0.3161)	0.0000	0.9999	0.4618 (0.3353)	1.3374	0.2108	0.2132
Belgium	0.0000 (0.3534)	0.0000	0.9999	0.3789 (0.3778)	1.0030	0.3546	0.1436
Canada	0.0000 (0.2651)	0.0000	0.9999	0.4831 (0.2769)	1.7447	0.1116	0.2334
Denmark	0.0000 (0.2231)	0.0000	0.9999	0.7468 (0.2351)	3.1760	0.0131*	0.5577
Finland	0.0000 (0.3072)	0.0000	0.9999	0.4016 (0.3238)	1.2403	0.2500	0.1613
France	0.0000 (0.2530)	0.0000	0.9999	0.6564 (0.2667)	2.4610	0.0393**	0.4309
Germany	0.0000 (0.3281)	0.0000	0.9999	0.2073 (0.3459)	0.5992	0.5656	0.0430
Greece	0.0000 (0.3131)	0.0000	0.9999	-0.3588 (0.3300)	-1.0873	0.3086	0.1288
Hong Kong	0.0000 (0.2710)	0.0000	0.9999	0.5892 (0.2857)	2.0624	0.0731*	0.3471
India	0.0000 (0.3231)	0.0000	0.9999	0.2685 (0.3406)	0.7884	0.4532	0.0721
Indonesia	0.0000 (0.2228)	0.0000	0.9999	0.7474 (0.2349)	3.1820	0.0130**	0.5586
Japan	0.0000 (0.3107)	0.0000	0.9999	0.3765 (0.3275)	1.1496	0.2835	0.1418
Malaysia	0.0000 (0.2275)	0.0000	0.9999	0.7348 (0.2398)	3.0638	0.0155**	0.5399
Netherlands	0.0000 (0.3533)	0.0000	0.9999	0.1308 (0.3747)	0.3490	0.7374	0.0171
Norway	0.0000 (0.1993)	0.0000	0.9999	0.8043 (0.2101	3.8284	0.0050***	0.6469
Philippines	0.0000 (0.4494)	0.0000	0.9999	0.4926 (0.5024)	0.9805	0.3991	0.2427
Portugal	0.0000 (0.3512)	0.0000	0.9999	0.3925 (0.3755)	1.0454	0.3361	0.1541
Qatar	0.0000 (0.4450)	0.0000	0.9999	-0.2220 (0.4875)	-0.4554	0.6724	0.0493
Saudi Arabia	0.0000 (0.2642)	0.0000	0.9999	0.3132 (0.2742)	1.1423	0.2756	0.0981
South Africa	0.0000 (0.3379)	0.0000	0.9999	0.4659 (0.3612)	1.2898	0.2446	0.2171
Spain	0.0000 (0.4495)	0.0000	0.9999	0.4923 (0.5025)	0.9796	0.3995	0.2424
Sri Lanka	0.0000 (0.2757)	0.0000	0.9999	0.4977 (0.2891)	1.7216	0.1192	0.2477
Sweden	0.0000 (0.3193)	0.0000	0.9999	0.4440 (0.3387	1.3109	0.2312	0.1971
Switzerland	0.0000 (0.2855)	0.0000	0.9999	0.4391 (0.2995	1.4662	0.1767	0.1928
Thailand	0.0000 (0.3790)	0.0000	0.9999	0.1228 (0.4052)	0.3030	0.7721	0.0151
UK	0.0000 (0.3175)	0.0000	0.9999	0.3224 (0.3347)	0.9634	0.3636	0.1040
USA	0.0000 (0.2441)	0.0000	0.9999	0.6858 (0.2573)	2.6649	0.0286**	0.4703

Table 16. Regression table: Dependent variable is the scaled subgraph centrality and the independent variable is the scaled employees (2012-13). *** : significant at 1%, **: at 5%, *: at 10%.

Countries	β_0	Tstat	Pvalue	β_1	Tstat	Pvalue	Rsquare
Australia	0.0000 (0.3151)	0.0000	0.9999	0.4672 (0.3342)	1.3981	0.2048	0.2183
Belgium	0.0000 (0.3673)	0.0000	0.9999	0.2735 (0.3927)	0.6964	0.5123	0.0748
Canada	0.0000 (0.2874)	0.0000	0.9999	0.3148 (0.3001)	1.0489	0.3189	0.0991
Denmark	0.0000 (0.2759)	0.0000	0.9999	0.5688 (0.2908)	1.9588	0.0862*	0.3235
Finland	0.0000 (0.2702)	0.0000	0.9999	0.5924 (0.2849)	2.0795	0.0712*	0.3509
France	0.0000 (0.2653)	0.0000	0.9999	0.6117 (0.2797)	2.1870	0.0602*	0.3742
Germany	0.0000 (0.2970)	0.0000	0.9999	0.4647 (0.3131)	1.4843	0.1760	0.2159
Greece	0.0000 (0.2995)	0.0000	0.9999	-0.4503 (0.3157)	-1.4263	0.1916	0.2027
Hong Kong	0.0000 (0.2565)	0.0000	0.9999	0.6444 (0.2704)	2.3836	0.0443**	0.4153
India	0.0000 (0.3352)	0.0000	0.9999	0.0384 (0.3533)	0.1086	0.9162	0.0015
Indonesia	0.0000 (0.2619)	0.0000	0.9999	0.6246 (0.2761)	2.2624	0.0535*	0.3902
Japan	0.0000 (0.2825)	0.0000	0.9999	0.5389 (0.2978)	1.8094	0.1080	0.2904
Malaysia	0.0000 (0.2666)	0.0000	0.9999	0.6066 (0.2811)	2.1583	0.0630*	0.3680
Netherlands	0.0000 (0.2573)	0.0000	0.9999	0.6918 (0.2729)	2.5349	0.0390**	0.4786
Norway	0.0000 (0.2046)	0.0000	0.9999	0.7924 (0.2157)	3.6742	0.0063***	0.6279
Philippines	0.0000 (0.4503	0.0000	0.9999	0.4894 (0.5035)	0.9719	0.4028	0.2395
Portugal	0.0000 (0.3797)	0.0000	0.9999	0.1068 (0.4059)	0.2632	0.801 2	0.0114
Qatar	- -						
Saudi Arabia	- -						
South Africa	0.0000 (0.3677)	0.0000	0.9999	0.2702 (0.3931)	0.6875	0.5174	0.0730
Spain	0.0000 (0.5163)	0.0000	0.9999	0.0218 (0.5772)	0.0377	0.9723	0.0004
Sri Lanka	0.0000 (0.2528)	0.0000	0.9999	0.6060 (0.2651)	2.2857	0.0481**	0.3673
Sweden	0.0000 (0.3294)	0.0000	0.9999	0.3814 (0.3494)	1.0917	0.3111	0.1455
Switzerland	0.0000 (0.2845)	0.0000	0.9999	0.4460 (0.2983)	1.4948	0.1692	0.1989
Thailand	0.0000 (0.2868)	0.0000	0.9999	0.6604 (0.3066)	2.1540	0.0747*	0.4361
UK	0.0000 (0.1957)	0.0000	0.9999	0.8122 (0.2063)	3.9377	0.0043	0.6597
USA	0.0000 (0.2775)	0.0000	0.9999	0.5618 (0.2925)	1.9210	0.0910*	0.3157

Table 17. Regression table: Dependent variable is the scaled subgraph centrality and the independent variable is the scaled number of market capitalization (2008-09). *** : significant at 1%, **: at 5%, *: at 10%.

Countries	β_0	Tstat	Pvalue	β_1	Tstat	Pvalue	Rsquare
Australia	0 (0.352)	0	1	0.1567 (0.3733)	0.4197	0.6874	0.0246
Belgium	0 (0.3801)	0	1	-0.0986 (0.4063)	-0.2427	0.8164	0.0098
Canada	0 (0.3025)	0	1	0.0432 (0.316)	0.1365	0.8942	0.0019
Switzerland	0 (0.3166)	0	1	-0.0884 (0.3321)	-0.2662	0.7962	0.0079
Germany	0 (0.2684)	0	1	0.6001 (0.2829)	2.1216	0.0667*	0.3601
Denmark	0 (0.2943)	0	1	0.48 (0.3102)	1.5476	0.1604	0.2304
Spain	0 (0.4885)	0	1	0.3248 (0.5461)	0.5949	0.5939	0.1055
Finland	- -						
France	0 (0.3093)	0	1	0.3871 (0.3261)	1.1872	0.2693	0.1498
UK	0 (0.335)	0	1	0.0502 (0.3532)	0.1421	0.8906	0.0026
Greece	0 (0.3324)	0	1	0.1341 (0.3504)	0.3828	0.7119	0.018
Hong Kong	0 (0.316)	0	1	0.3358 (0.3331)	1.0081	0.343	0.1128
Indonesia	0 (0.2908)	0	1	0.4985 (0.3065)	1.6265	0.1426	0.2486
India	0 (0.2755)	0	1	0.5704 (0.2904)	1.9642	0.0852*	0.3254
Japan	0 (0.3337)	0	1	0.1037 (0.3517)	0.2949	0.7757	0.0108
Sri Lanka	0 (0.3019)	0	1	-0.3135 (0.3166)	-0.9904	0.3479	0.0983
Malaysia	0 (0.2642)	0	1	0.6162 (0.2785)	2.2126	0.0579*	0.3797
Netherland	0 (0.3212)	0	1	0.4335 (0.3407)	1.2727	0.2438	0.1879
Norway	0 (0.2981)	0	1	0.4586 (0.3142)	1.4594	0.1826	0.2103
Philippines	0 (0.4278)	0	1	0.5603 (0.4783)	1.1716	0.326	0.3139
Portugal	0 (0.2959)	0	1	0.6325 (0.3163)	1.9999	0.0925*	0.4
Qatar	0 (0.389)	0	1	0.5234 (0.4261)	1.2284	0.2867	0.274
Saudi Arabia	0 (0.2718)	0	1	0.2143 (0.282)	0.76	0.462	0.046
Sweden	0 (0.3416)	0	1	0.2855 (0.3623)	0.7882	0.4565	0.0816
Thailand	0 (0.368)	0	1	0.2677 (0.3934)	0.6804	0.5217	0.0717
USA	0 (0.3311)	0	1	0.1612 (0.349)	0.4619	0.6565	0.026
South Africa	0 (0.3794)	0	1	-0.1149 (0.4056)	-0.2833	0.7866	0.0132

Table 18. Regression table: Dependent variable is the scaled subgraph centrality and the independent variable is the scaled number of revenue (2008-09). *** : significant at 1%, **: at 5%, *: at 10%.

Countries	β_0	Tstat	Pvalue	β_1	Tstat	Pvalue	Rsquare
Australia	0 (0.3372)	0	1	0.3238 (0.3577)	0.9054	0.3954	0.1049
Belgium	0 (0.3698)	0	1	-0.2506 (0.3953)	-0.6339	0.5496	0.0628
Canada	0 (0.2845)	0	1	0.3428 (0.2971)	1.1537	0.2755	0.1175
Switzerland	0 (0.3163)	0	1	0.0991 (0.3317)	0.2988	0.772	0.0099
Germany	0 (0.2736)	0	1	0.5785 (0.2884)	2.0059	0.0798*	0.3347
Denmark	0 (0.268)	0	1	0.6015 (0.2825)	2.1297	0.0659*	0.3618
Spain	0 (0.3636)	0	1	0.7102 (0.4065)	1.7472	0.179	0.5044
Finland	- -						
France	0 (0.2438)	0	1	0.687 (0.257)	2.6734	0.0283**	0.4719
UK	0 (0.3305)	0	1	0.1715 (0.3484)	0.4924	0.6358	0.0295
Greece	0 (0.3077)	0	1	-0.3987 (0.3243)	-1.2295	0.2539	0.159
Hong Kong	0 (0.2925)	0	1	0.4898 (0.3083)	1.5889	0.1508	0.2399
Indonesia	0 (0.268)	0	1	0.6015 (0.2825)	2.1292	0.0659*	0.3618
India	0 (0.3027)	0	1	0.431 (0.3191)	1.3507	0.2138	0.1857
Japan	0 (0.3227)	0	1	0.2735 (0.3401)	0.8041	0.4447	0.0748
Sri Lanka	0 (0.3138)	0	1	-0.1594 (0.3291)	-0.4843	0.6398	0.0254
Malaysia	0 (0.2645)	0	1	0.6153 (0.2788)	2.2074	0.0584*	0.3786
Netherland	0 (0.3445)	0	1	0.2565 (0.3654)	0.7022	0.5053	0.0658
Norway	0 (0.2434)	0	1	0.6883 (0.2566)	2.6831	0.0278**	0.4737
Philippines	0 (0.4836)	0	1	0.3511 (0.5406)	0.6495	0.5624	0.1233
Portugal	0 (0.2686)	0	1	0.711 (0.2872)	2.4762	0.0481**	0.5055
Qatar	0 (0.3953)	0	1	-0.5003 (0.433)	-1.1555	0.3123	0.2503
Saudi Arabia	0 (0.2741)	0	1	0.1711 (0.2845)	0.6015	0.5588	0.0293
Sweden	0 (0.3043)	0	1	0.5209 (0.3227)	1.6142	0.1506	0.2713
Thailand	0 (0.374)	0	1	0.2026 (0.3998)	0.5067	0.6305	0.0411
USA	0 (0.3316)	0	1	0.152 (0.3495)	0.435	0.6752	0.0231
South Africa	0 (0.3237)	0	1	0.5308 (0.346)	1.534	0.176	0.2818

Table 19. Regression table: Dependent variable is the scaled subgraph centrality and the independent variable is the scaled number of employees (2008-09). *** : significant at 1%, **: at 5%, *: at 10%.

Countries	β_0	Tstat	Pvalue	β_1	Tstat	Pvalue	Rsquare
Australia	0 (0.3386)	0.00000	1.00000	0.3113 (0.3592)	0.8667	0.4148	0.0969
Belgium	0 (0.3809)	0.00000	1.00000	-0.071 (0.4072)	-0.1743	0.8674	0.005
Canada	0 (0.2927)	0.00000	1.00000	0.2559 (0.3057)	0.837	0.4221	0.0655
Switzerland	0 (0.3091)	0.00000	1.00000	0.2322 (0.3242)	0.7163	0.492	0.0539
Germany	0 (0.2854)	0.00000	1.00000	0.5256 (0.3008)	1.7472	0.1187	0.2762
Denmark	0 (0.3052)	0.00000	1.00000	0.4146 (0.3217)	1.2887	0.2335	0.1719
Spain	0 (0.4001)	0.00000	1.00000	0.6322 (0.4473)	1.4134	0.2524	0.3997
Finland	- -						
France	0 (0.2508)	0.00000	1.00000	0.6641 (0.2643)	2.5127	0.0362**	0.4411
UK	0 (0.2385)	0.00000	1.00000	0.7032 (0.2514)	2.7974	0.0233**	0.4945
Greece	0 (0.3354)	0.00000	1.00000	-0.0079 (0.3535)	-0.0224	0.9827	0.0001
Hong Kong	0 (0.2853)	0.00000	1.00000	0.5257 (0.3008)	1.748	0.1186	0.2764
Indonesia	0 (0.2682)	0.00000	1.00000	0.6006 (0.2827)	2.1249	0.0663*	0.3608
India	0 (0.3089)	0.00000	1.00000	0.3898 (0.3256)	1.1972	0.2655	0.1519
Japan	0 (0.292)	0.00000	1.00000	0.4919 (0.3078)	1.5978	0.1488	0.2419
Sri Lanka	0 (0.3024)	0.00000	1.00000	0.3075 (0.3172)	0.9695	0.3576	0.0946
Malaysia	0 (0.2737)	0.00000	1.00000	0.5781 (0.2885)	2.0037	0.0801*	0.3342
Netherland	0 (0.3095)	0.00000	1.00000	0.4955 (0.3283)	1.5091	0.175	0.2455
Norway	0 (0.2407)	0.00000	1.00000	0.6963 (0.2538)	2.7438	0.0253**	0.4848
Philippines	0 (0.4851)	0.00000	1.00000	0.3429 (0.5423)	0.6323	0.5721	0.1176
Portugal	0 (0.3119)	0.00000	1.00000	0.5769 (0.3335)	1.7299	0.1344	0.3328
Qatar	- -						
Saudi Arabia	- -						
Sweden	0 (0.3182)	0.00000	1.00000	0.4499 (0.3375)	1.3329	0.2243	0.2024
Thailand	0 (0.3585)	0.00000	1.00000	-0.3448 (0.3832)	-0.8997	0.4029	0.1189
USA	0 (0.2752)	0.00000	1.00000	0.5716 (0.2901)	1.9705	0.0843*	0.3268
ZAF	0 (0.3663)	0.00000	1.00000	0.2825 (0.3916)	0.7213	0.4979	0.0798

Table 20. Regression table: Dependent variable is the scaled node strength and the independent variable is the scaled market capitalization (2015-16). *** : significant at 1%, **: at 5%, *: at 10%.

Countries	β_0	Tstat	Pvalue	β_1	Tstat	Pvalue	Rsquare
Australia	0.0000 (0.3473)	0.0000	0.9999	0.2234 (0.3684)	0.6065	0.5633	0.0499
Belgium	0.0000 (0.3704)	0.0000	0.9999	0.2431 (0.3960)	0.6140	0.5617	0.0591
Canada	0.0000 (0.3001)	0.0000	0.9999	0.1328 (0.3134)	0.4238	0.6807	0.0176
Denmark	0.0000 (0.2657)	0.0000	0.9999	0.6102 (0.2801)	2.1785	0.0610*	0.3723
Finland	0.0000 (0.2203)	0.0000	0.9999	0.7541 (0.2322)	3.2480	0.0117**	0.5687
France	0.0000 (0.2385)	0.0000	0.9999	0.7030 (0.2514)	2.7960	0.0233**	0.4942
Germany	0.0000 (0.2496)	0.0000	0.9999	0.6682 (0.2631)	2.5400	0.0347**	0.4464
Greece	0.0000 (0.1626)	0.0000	0.9999	-0.8747 (0.1713)	-5.1050	0.0009***	0.7651
Hong Kong	0.0000 (0.3019)	0.0000	0.9999	0.4356 (0.3182)	1.3687	0.2083	0.1897
India	0.0000 (0.3322)	0.0000	0.9999	0.1370 (0.3502)	0.3913	0.7058	0.0188
Indonesia	0.0000 (0.2330)	0.0000	0.9999	0.7195 (0.2456)	2.9300	0.0190**	0.5176
Japan	0.0000 (0.3237)	0.0000	0.9999	0.2619 (0.3412)	0.7677	0.4647	0.0686
Malaysia	0.0000 (0.2702)	0.0000	0.9999	0.5923 (0.2849)	2.0794	0.0712*	0.3509
Netherlands	0.0000 (0.3314)	0.0000	0.9999	0.3673 (0.3515)	1.0450	0.3308	0.1349
Norway	0.0000 (0.3035)	0.0000	0.9999	0.4257 (0.3199)	1.3306	0.2200	0.1812
Philippines	0.0000 (0.1545)	0.0000	0.9999	0.9542 (0.1727)	5.5241	0.0117**	0.9105
Portugal	0.0000 (0.3164)	0.0000	0.9999	0.5600 (0.3382)	1.6556	0.1489	0.3136
Qatar	0.0000 (0.3769)	0.0000	0.9999	0.5640 (0.4129)	1.3659	0.2437	0.3181
Saudi Arabia	0.0000 (0.2673)	0.0000	0.9999	0.2772 (0.2774)	0.9995	0.3373	0.0768
South Africa	0.0000 (0.3731)	0.0000	0.9999	-0.2132 (0.3989)	-0.5344	0.6123	0.0454
Spain	0.0000 (0.5108)	0.0000	0.9999	0.1468 (0.5711)	0.2570	0.8138	0.0215
Sri Lanka	0.0000 (0.2623)	0.0000	0.9999	0.5645 (0.2752)	2.0515	0.0704*	0.3186
Sweden	0.0000 (0.2826)	0.0000	0.9999	0.6093 (0.2997)	2.0329	0.0816*	0.3712
Switzerland	0.0000 (0.2834)	0.0000	0.9999	0.4524 (0.2973)	1.5218	0.1624	0.2047
Thailand	0.0000 (0.3152)	0.0000	0.9999	0.5644 (0.3370)	1.6746	0.1450	0.3185
UK	0.0000 (0.3103)	0.0000	0.9999	0.3798 (0.3271)	1.1614	0.2790	0.1443
USA	0.0000 (0.2747)	0.0000	0.9999	0.5737 (0.2896)	1.9812	0.0829*	0.3292

Table 21. Regression table: Dependent variable is the scaled node strength and the independent variable is the scaled revenue (2015-16). *** : significant at 1%, **: at 5%, *: at 10%.

Countries	β_0	Tstat	Pvalue	β_1	Tstat	Pvalue	Rsquare
Australia	0.0000 (0.3121)	0.0000	0.9999	0.4828 (0.3310)	1.4586	0.1880	0.2331
Belgium	0.0000 (0.2279)	0.0000	0.9999	0.8023 (0.2437)	3.2925	0.0166**	0.6437
Canada	0.0000 (0.2890)	0.0000	0.9999	0.2979 (0.3019)	0.9869	0.3470	0.0887
Denmark	0.0000 (0.2800)	0.0000	0.9999	0.5507 (0.2951)	1.8663	0.0990*	0.3033
Finland	0.0000 (0.2975)	0.0000	0.9999	0.4621 (0.3135)	1.4738	0.1788	0.2135
France	0.0000 (0.2346)	0.0000	0.9999	0.7148 (0.2472)	2.8910	0.0202**	0.5109
Germany	0.0000 (0.3197)	0.0000	0.9999	0.3025 (0.3370)	0.8977	0.3956	0.0915
Greece	0.0000 (0.3071)	0.0000	0.9999	-0.4021 (0.3237)	-1.2423	0.2493	0.1617
Hong Kong	0.0000 (0.2848)	0.0000	0.9999	0.5284 (0.3002)	1.7604	0.1164	0.2792
India	0.0000 (0.3145)	0.0000	0.9999	0.3474 (0.3315)	1.0479	0.3253	0.1207
Indonesia	0.0000 (0.2322)	0.0000	0.9999	0.7217 (0.2447)	2.9485	0.0185**	0.5208
Japan	0.0000 (0.3126)	0.0000	0.9999	0.3622 (0.3295)	1.0991	0.3037	0.1312
Malaysia	0.0000 (0.2725)	0.0000	0.9999	0.5831 (0.2872)	2.0302	0.0768*	0.3400
Netherlands	0.0000 (0.3418)	0.0000	0.9999	0.2826 (0.3626)	0.7795	0.4612	0.0799
Norway	0.0000 (0.2242)	0.0000	0.9999	0.7438 (0.2363)	3.1474	0.0137**	0.5532
Philippines	0.0000 (0.4569)	0.0000	0.9999	0.4659 (0.5109)	0.9120	0.4290	0.2171
Portugal	0.0000 (0.2672)	0.0000	0.9999	0.7143 (0.2857)	2.5002	0.0465**	0.5103
Qatar	0.0000 (0.4534)	0.0000	0.9999	-0.1144 (0.4967)	-0.2303	0.8291	0.0131
Saudi Arabia	- -						
South Africa	0.0000 (0.3142)	0.0000	0.9999	0.5684 (0.3359)	1.6922	0.1416	0.3231
Spain	0.0000 (0.5105)	0.0000	0.9999	0.1511 (0.5707)	0.2647	0.8084	0.0228
Sri Lanka	0.0000 (0.2373)	0.0000	0.9999	0.6653 (0.2489)	2.6736	0.0255**	0.4427
Sweden	0.0000 (0.2920)	0.0000	0.9999	0.5730 (0.3098)	1.8499	0.1068	0.3284
Switzerland	0.0000 (0.2633)	0.0000	0.9999	0.5601 (0.2761)	2.0281	0.0732*	0.3137
Thailand	0.0000 (0.3586)	0.0000	0.9999	0.3439 (0.3834)	0.8970	0.4043	0.1182
UK	0.0000 (0.3353)	0.0000	0.9999	-0.0299 (0.3534)	-0.0847	0.9346	0.0008
USA	0.0000 (0.2678)	0.0000	0.9999	0.6020 (0.2823)	2.1326	0.0655*	0.3624

Table 22. Regression table: Dependent variable is the scaled node strength and the independent variable is the scaled number of employees (2015-16). *** : significant at 1%, **: at 5%, *: at 10%.

Countries	β_0	Tstat	Pvalue	β_1	Tstat	Pvalue	Rsquare
Australia	0.0000 (0.3026)	0.0000	0.9999	0.5279 (0.3210)	1.6446	0.1440	0.2787
Belgium	0.0000 (0.2578)	0.0000	0.9999	0.7378 (0.2756)	2.6773	0.0367**	0.5444
Canada	0.0000 (0.2923)	0.0000	0.9999	0.2608 (0.3053)	0.8544	0.4129	0.0680
Denmark	0.0000 (0.3146)	0.0000	0.9999	0.3467 (0.3316)	1.0455	0.3264	0.1202
Finland	0.0000 (0.2747)	0.0000	0.9999	0.5739 (0.2895)	1.9822	0.0828*	0.3294
France	0.0000 (0.1509)	0.0000	0.9999	0.8930 (0.1591)	5.6137	0.0005***	0.7975
Germany	0.0000 (0.2794)	0.0000	0.9999	0.5534 (0.2945)	1.8791	0.0970*	0.3062
Greece	0.0000 (0.1549)	0.0000	0.9999	-0.8870 (0.1633)	-5.4327	0.0006***	0.7867
Hong Kong	0.0000 (0.2415)	0.0000	0.9999	0.6939 (0.2546)	2.7253	0.0260**	0.4814
India	0.0000 (0.3353)	0.0000	0.9999	0.0283 (0.3534)	0.0802	0.9381	0.0008
Indonesia	0.0000 (0.2799)	0.0000	0.9999	0.5508 (0.2951)	1.8666	0.0989*	0.3034
Japan	0.0000 (0.2914)	0.0000	0.9999	0.4953 (0.3071)	1.6126	0.1455	0.2453
Malaysia	0.0000 (0.2953)	0.0000	0.9999	0.4741 (0.3113)	1.5230	0.1663	0.2248
Netherlands	0.0000 (0.2239)	0.0000	0.9999	0.7779 (0.2375)	3.2750	0.0136**	0.6051
Norway	0.0000 (0.2765)	0.0000	0.9999	0.5662 (0.2914)	1.9427	0.0880*	0.3205
Philippines	0.0000 (0.4712)	0.0000	0.9999	0.4093 (0.5268)	0.7769	0.4938	0.1675
Portugal	0.0000 (0.3194)	0.0000	0.9999	0.5481 (0.3415)	1.6053	0.1595	0.3005
Qatar	- -						
Saudi Arabia	- -						
South Africa	0.0000 (0.3770)	0.0000	0.9999	0.1588 (0.4031)	0.3940	0.7072	0.0252
Spain	0.0000 (0.4825)	0.0000	0.9999	0.3561 (0.5395)	0.6601	0.5563	0.1268
Sri Lanka	0.0000 (0.1813)	0.0000	0.9999	0.8213 (0.1901)	4.3196	0.0019***	0.6746
Sweden	0.0000 (0.3130)	0.0000	0.9999	0.4781 (0.3320)	1.4402	0.1930	0.2286
Switzerland	0.0000 (0.2698)	0.0000	0.9999	0.5285 (0.2830)	1.8676	0.0947*	0.2793
Thailand	- -						
UK	0.0000 (0.2523)	0.0000	0.9999	0.6590 (0.2659)	2.4780	0.0382**	0.4342
USA	0.0000 (0.2492)	0.0000	0.9999	0.6692 (0.2627)	2.5470	0.0343**	0.4478

Table 23. Regression table: Dependent variable is the scaled node strength and the independent variable is the scaled market capitalization (2012-13). *** : significant at 1%, **: at 5%, *: at 10%.

Countries	β_0	Tstat	Pvalue	β_1	Tstat	Pvalue	Rsquare
Australia	0.0000 (0.3329)	0.0000	0.9999	0.3565 (0.3531)	1.0096	0.3463	0.1271
Belgium	0.0000 (0.3684)	0.0000	0.9999	0.2634 (0.3938)	0.6689	0.5284	0.0694
Canada	0.0000 (0.2727)	0.0000	0.9999	0.4347 (0.2848)	1.5263	0.1579	0.1889
Denmark	0.0000 (0.2510)	0.0000	0.9999	0.6632 (0.2646)	2.5066	0.0366**	0.4399
Finland	0.0000 (0.2807)	0.0000	0.9999	0.5476 (0.2958)	1.8509	0.1013	0.2998
France	0.0000 (0.2792)	0.0000	0.9999	0.5540 (0.2943)	1.8820	0.0966*	0.3069
Germany	0.0000 (0.2822)	0.0000	0.9999	0.5404 (0.2975)	1.8168	0.1068	0.2921
Greece	0.0000 (0.2916)	0.0000	0.9999	-0.4939 (0.3074)	-1.6068	0.1468	0.2440
Hong Kong	0.0000 (0.2958)	0.0000	0.9999	0.4713 (0.3118)	1.5113	0.1692	0.2221
India	0.0000 (0.3278)	0.0000	0.9999	-0.2116 (0.3455)	-0.6125	0.5572	0.0448
Indonesia	0.0000 (0.2480)	0.0000	0.9999	0.6733 (0.2614)	2.5756	0.0328**	0.4533
Japan	0.0000 (0.3218)	0.0000	0.9999	0.2819 (0.3392)	0.8312	0.4300	0.0795
Malaysia	0.0000 (0.2418)	0.0000	0.9999	0.6930 (0.2549)	2.7188	0.0263**	0.4802
Netherlands	0.0000 (0.3417)	0.0000	0.9999	0.2838 (0.3624)	0.7832	0.4592	0.0806
Norway	0.0000 (0.2859)	0.0000	0.9999	0.5230 (0.3014)	1.7354	0.1209	0.2735
Philippines	0.0000 (0.2860)	0.0000	0.9999	0.8327 (0.3197)	2.6046	0.0801*	0.6934
Portugal	0.0000 (0.3199)	0.0000	0.9999	0.5462 (0.3420)	1.5973	0.1613	0.2984
Qatar	0.0000 (0.3442)	0.0000	0.9999	0.6567 (0.3771)	1.7415	0.1566	0.4312
Saudi Arabia	0.0000 (0.2613)	0.0000	0.9999	0.3427 (0.2712)	1.2638	0.2303	0.1175
South Africa	0.0000 (0.3815)	0.0000	0.9999	0.0449 (0.4078)	0.1102	0.9159	0.0020
Spain	0.0000 (0.3440)	0.0000	0.9999	0.7458 (0.3846)	1.9390	0.1479	0.5562
Sri Lanka	0.0000 (0.3125)	0.0000	0.9999	0.1830 (0.3277)	0.5584	0.5902	0.0335
Sweden	0.0000 (0.3301)	0.0000	0.9999	0.3770 (0.3501)	1.0769	0.3173	0.1421
Switzerland	0.0000 (0.3045)	0.0000	0.9999	0.2863 (0.3194)	0.8963	0.3935	0.0819
Thailand	0.0000 (0.3113)	0.0000	0.9999	0.5791 (0.3328)	1.7400	0.1325	0.3354
UK	0.0000 (0.2793)	0.0000	0.9999	0.5535 (0.2945)	1.8798	0.0969	0.3064
USA	0.0000 (0.2817)	0.0000	0.9999	0.5426 (0.2970)	1.8270	0.1051	0.2944

Table 24. Regression table: Dependent variable is the scaled node strength and the independent variable is the scaled revenue (2012-13). *** : significant at 1%, **: at 5%, *: at 10%.

Countries	β_0	Tstat	Pvalue	β_1	Tstat	Pvalue	Rsquare
Australia	0.0000 (0.3178)	0.0000	0.9999	0.4525 (0.3371)	1.3425	0.2213	0.2048
Belgium	0.0000 (0.3542)	0.0000	0.9999	0.3735 (0.3787)	0.9862	0.3621	0.1395
Canada	0.0000 (0.2694)	0.0000	0.9999	0.4563 (0.2814)	1.6218	0.1359	0.2082
Denmark	0.0000 (0.2594)	0.0000	0.9999	0.6341 (0.2734)	2.3194	0.0490**	0.4021
Finland	0.0000 (0.3056)	0.0000	0.9999	0.4122 (0.3221)	1.2798	0.2365	0.1699
France	0.0000 (0.2517)	0.0000	0.9999	0.6611 (0.2653)	2.4923	0.0374**	0.4371
Germany	0.0000 (0.3274)	0.0000	0.9999	0.2172 (0.3451)	0.6293	0.5467	0.0472
Greece	0.0000 (0.3120)	0.0000	0.9999	-0.3668 (0.3289)	-1.1153	0.2971	0.1346
Hong Kong	0.0000 (0.2704)	0.0000	0.9999	0.5917 (0.2850)	2.0758	0.0716*	0.3501
India	0.0000 (0.3245)	0.0000	0.9999	0.2527 (0.3421)	0.7388	0.4811	0.0639
Indonesia	0.0000 (0.2284)	0.0000	0.9999	0.7323 (0.2408)	3.0417	0.0160**	0.5363
Japan	0.0000 (0.3113)	0.0000	0.9999	0.3719 (0.3282)	1.1333	0.2899	0.1383
Malaysia	0.0000 (0.2428)	0.0000	0.9999	0.6900 (0.2559)	2.6961	0.0272**	0.4761
Netherlands	0.0000 (0.3516)	0.0000	0.9999	0.1621 (0.3730)	0.4347	0.6769	0.0263
Norway	0.0000 (0.2273)	0.0000	0.9999	0.7354 (0.2396)	3.0699	0.0154**	0.5409
Philippines	0.0000 (0.4486)	0.0000	0.9999	0.4952 (0.5016)	0.9873	0.3963	0.2453
Portugal	0.0000 (0.3442)	0.0000	0.9999	0.4332 (0.3680)	1.1772	0.2837	0.1876
Qatar	0.0000 (0.4428)	0.0000	0.9999	-0.2428 (0.4850)	-0.5006	0.6430	0.0590
Saudi Arabia	0.0000 (0.2656)	0.0000	0.9999	0.2975 (0.2756)	1.0793	0.3017	0.0885
South Africa	0.0000 (0.3305)	0.0000	0.9999	0.5009 (0.3533)	1.4177	0.2061	0.2509
Spain	0.0000 (0.4430)	0.0000	0.9999	0.5139 (0.4953)	1.0377	0.3757	0.2641
Sri Lanka	0.0000 (0.2810)	0.0000	0.9999	0.4673 (0.2947)	1.5856	0.1473	0.2183
Sweden	0.0000 (0.3229)	0.0000	0.9999	0.4231 (0.3425)	1.2354	0.2565	0.1790
Switzerland	0.0000 (0.2858)	0.0000	0.9999	0.4372 (0.2998)	1.4583	0.1788	0.1911
Thailand	0.0000 (0.3786)	0.0000	0.9999	0.1309 (0.4047)	0.3234	0.7573	0.0171
UK	0.0000 (0.3171)	0.0000	0.9999	0.3255 (0.3343)	0.9737	0.3587	0.1059
USA	0.0000 (0.2378)	0.0000	0.9999	0.7053 (0.2506)	2.8144	0.0227**	0.4975

Table 25. Regression table: Dependent variable is the scaled node strength and the independent variable is the scaled number of employees (2012-13). *** : significant at 1%, **: at 5%, *: at 10%.

Countries	β_0	Tstat	Pvalue	β_1	Tstat	Pvalue	Rsquare
Australia	0.0000 (0.3131)	0.0000	0.9999	0.4774 (0.3321)	1.4375	0.1937	0.2279
Belgium	0.0000 (0.3669)	0.0000	0.9999	0.2771 (0.3923)	0.7065	0.5064	0.0768
Canada	0.0000 (0.2919)	0.0000	0.9999	0.2658 (0.3049)	0.8719	0.4037	0.0707
Denmark	0.0000 (0.2910)	0.0000	0.9999	0.4974 (0.3067)	1.6216	0.1435	0.2474
Finland	0.0000 (0.2768)	0.0000	0.9999	0.5648 (0.2918)	1.9358	0.0889*	0.3190
France	0.0000 (0.2667)	0.0000	0.9999	0.6065 (0.2811)	2.1577	0.0630*	0.3679
Germany	0.0000 (0.3011)	0.0000	0.9999	0.4409 (0.3173)	1.3893	0.2022	0.1944
Greece	0.0000 (0.2971)	0.0000	0.9999	-0.4638 (0.3132)	-1.4808	0.1769	0.2151
Hong Kong	0.0000 (0.2556)	0.0000	0.9999	0.6475 (0.2694)	2.4034	0.0430**	0.4193
India	0.0000 (0.3354)	0.0000	0.9999	-0.0178 (0.3535)	-0.0505	0.9610	0.0003
Indonesia	0.0000 (0.2677)	0.0000	0.9999	0.6027 (0.2821)	2.1362	0.0652*	0.3632
Japan	0.0000 (0.2845)	0.0000	0.9999	0.5296 (0.2999)	1.7658	0.1154	0.2804
Malaysia	0.0000 (0.2796)	0.0000	0.9999	0.5521 (0.2948)	1.8731	0.0979*	0.3049
Netherlands	0.0000 (0.2595)	0.0000	0.9999	0.6852 (0.2753)	2.4892	0.0416**	0.4695
Norway	0.0000 (0.2252)	0.0000	0.9999	0.7412 (0.2374)	3.1226	0.0142**	0.5493
Philippines	0.0000 (0.4502)	0.0000	0.9999	0.4898 (0.5034)	0.9729	0.4023	0.2399
Portugal	0.0000 (0.3730)	0.0000	0.9999	0.2144 (0.3988)	0.5375	0.6102	0.0459
Qatar	- -						
Saudi Arabia	- -						
South Africa	0.0000 (0.3621)	0.0000	0.9999	0.3180 (0.3871)	0.8217	0.4427	0.1011
Spain	0.0000 (0.5158)	0.0000	0.9999	0.0489 (0.5767)	0.0848	0.9378	0.0024
Sri Lanka	0.0000 (0.2602)	0.0000	0.9999	0.5744 (0.2729)	2.1050	0.0646*	0.3299
Sweden	0.0000 (0.3324)	0.0000	0.9999	0.3608 (0.3525)	1.0234	0.3402	0.1301
Switzerland	0.0000 (0.2873)	0.0000	0.9999	0.4274 (0.3013)	1.4184	0.1897	0.1827
Thailand	0.0000 (0.2876)	0.0000	0.9999	0.6578 (0.3075)	2.1394	0.0762*	0.4327
UK	0.0000 (0.2042)	0.0000	0.9999	0.7934 (0.2152)	3.6865	0.0062***	0.6295
USA	0.0000 (0.2744)	0.0000	0.9999	0.5752 (0.2892)	1.9891	0.0819*	0.3309

Table 26. Regression table: Dependent variable is the scaled node strength and the independent variable is the scaled market capitalization (2008-09). *** : significant at 1%, **: at 5%, *: at 10%.

Countries	β_0	Tstat	Pvalue	β_1	Tstat	Pvalue	Rsquare
Australia	0.0000 (0.3525)	0.0000	0.9999	0.1464 (0.3739)	0.3915	0.7071	0.0214
Belgium	0.0000 (0.3796)	0.0000	0.9999	-0.1095 (0.4058)	-0.2698	0.7964	0.0120
Canada	0.0000 (0.3025)	0.0000	0.9999	0.0448 (0.3159)	0.1417	0.8902	0.0020
Denmark	0.0000 (0.3047)	0.0000	0.9999	0.4180 (0.3212)	1.3016	0.2293	0.1748
Finland	- -						
France	0.0000 (0.3093)	0.0000	0.9999	0.3868 (0.3260)	1.1865	0.2695	0.1496
Germany	0.0000 (0.2656)	0.0000	0.9999	0.6107 (0.2800)	2.1814	0.0607*	0.3730
Greece	0.0000 (0.3327)	0.0000	0.9999	0.1270 (0.3507)	0.3621	0.7266	0.0161
Hong Kong	0.0000 (0.3156)	0.0000	0.9999	0.3383 (0.3327)	1.0169	0.3390	0.1145
India	0.0000 (0.2759)	0.0000	0.9999	0.5688 (0.2908)	1.9563	0.0861*	0.3236
Indonesia	0.0000 (0.2900)	0.0000	0.9999	0.5022 (0.3057)	1.6426	0.1391	0.2522
Japan	0.0000 (0.3338)	0.0000	0.9999	0.0971 (0.3519)	0.2759	0.7896	0.0094
Malaysia	0.0000 (0.2723)	0.0000	0.9999	0.5837 (0.2871)	2.0332	0.0765*	0.3407
Netherlands	0.0000 (0.3137)	0.0000	0.9999	0.4744 (0.3327)	1.4258	0.1970	0.2251
Norway	0.0000 (0.3022)	0.0000	0.9999	0.4336 (0.3186)	1.3612	0.2106	0.1880
Philippines	0.0000 (0.4233)	0.0000	0.9999	0.5727 (0.4733)	1.2099	0.3129	0.3280
Portugal	0.0000 (0.3132)	0.0000	0.9999	0.5721 (0.3348)	1.7085	0.1384	0.3273
Qatar	0.0000 (0.3909)	0.0000	0.9999	0.5163 (0.4282)	1.2058	0.2943	0.2666
Saudi Arabia	0.0000 (0.2718)	0.0000	0.9999	0.2124 (0.2821)	0.7530	0.4660	0.0451
South Africa	0.0000 (0.3802)	0.0000	0.9999	-0.0949 (0.4064)	-0.2334	0.8232	0.0090
Spain	0.0000 (0.4857)	0.0000	0.9999	0.3396 (0.5430)	0.6253	0.5761	0.1153
Sri Lanka	0.0000 (0.3164)	0.0000	0.9999	-0.0942 (0.3319)	- 0.2838	0.7829	0.0089
Sweden	0.0000 (0.3422)	0.0000	0.9999	0.2786 (0.3630)	0.7676	0.4678	0.0776
Switzerland	0.0000 (0.3176)	0.0000	0.9999	-0.0387 (0.3331)	-0.1162	0.9100	0.0015
Thailand	0.0000 (0.3677)	0.0000	0.9999	0.2704 (0.3930)	0.6879	0.5172	0.0731
UK	0.0000 (0.3350)	0.0000	0.9999	0.0473 (0.3532)	0.1339	0.8968	0.0022
USA	0.0000 (0.3317)	0.0000	0.9999	0.1480 (0.3497)	0.4232	0.6833	0.0219

Table 27. Regression table: Dependent variable is the scaled node strength and the independent variable is the scaled revenue (2008-09). *** : significant at 1%, **: at 5%, *: at 10%.

Countries	β_0	Tstat	Pvalue	β_1	Tstat	Pvalue	Rsquare
Australia	0.0000 (0.3370)	0.0000	0.9999	0.3252 (0.3574)	0.9099	0.3931	0.1058
Belgium	0.0000 (0.3685)	0.0000	0.9999	-0.2626 (0.3939)	-0.6665	0.5299	0.0689
Canada	0.0000 (0.2860)	0.0000	0.9999	0.3282 (0.2987)	1.0987	0.2976	0.1077
Denmark	0.0000 (0.2880)	0.0000	0.9999	0.5126 (0.3036)	1.6887	0.1297	0.2628
Finland	- -						
France	0.0000 (0.2447)	0.0000	0.9999	0.6838 (0.2580)	2.6508	0.0292**	0.4676
Germany	0.0000 (0.2735)	0.0000	0.9999	0.5788 (0.2883)	2.0075	0.0796*	0.3350
Greece	0.0000 (0.3074)	0.0000	0.9999	-0.4003 (0.3240)	-1.2354	0.2517	0.1602
Hong Kong	0.0000 (0.2935)	0.0000	0.9999	0.4842 (0.3093)	1.5653	0.1561	0.2345
India	0.0000 (0.3027)	0.0000	0.9999	0.4309 (0.3191)	1.3504	0.2138	0.1856
Indonesia	0.0000 (0.2716)	0.0000	0.9999	0.5868 (0.2863)	2.0498	0.0745*	0.3444
Japan	0.0000 (0.3230)	0.0000	0.9999	0.2698 (0.3404)	0.7926	0.4509	0.0728
Malaysia	0.0000 (0.2738)	0.0000	0.9999	0.5775 (0.2886)	2.0007	0.0804*	0.3335
Netherlands	0.0000 (0.3385)	0.0000	0.9999	0.3123 (0.3591)	0.8698	0.4132	0.0975
Norway	0.0000 (0.2499)	0.0000	0.9999	0.6671 (0.2634)	2.5331	0.0351**	0.4451
Philippines	0.0000 (0.4807)	0.0000	0.9999	0.3651 (0.5375)	0.6793	0.5457	0.1333
Portugal	0.0000 (0.2944)	0.0000	0.9999	0.6368 (0.3148)	2.0233	0.0895*	0.4056
Qatar	0.0000 (0.3951)	0.0000	0.9999	-0.5005 (0.4329)	-1.1564	0.3119	0.2505
Saudi Arabia	0.0000 (0.2743)	0.0000	0.9999	0.1671 (0.2846)	0.5870	0.5681	0.0279
South Africa	0.0000 (0.3195)	0.0000	0.9999	0.5477 (0.3416)	1.6036	0.1599	0.3000
Spain	0.0000 (0.3586)	0.0000	0.9999	0.7196 (0.4009)	1.7951	0.1705	0.5179
Sri Lanka	0.0000 (0.3175)	0.0000	0.9999	0.0435 (0.3330)	0.1307	0.8989	0.0019
Sweden	0.0000 (0.3060)	0.0000	0.9999	0.5125 (0.3246)	1.5791	0.1583	0.2627
Switzerland	0.0000 (0.3146)	0.0000	0.9999	0.1413 (0.3300)	0.4283	0.6785	0.0200
Thailand	0.0000 (0.3740)	0.0000	0.9999	0.2023 (0.3998)	0.5059	0.6309	0.0409
UK	0.0000 (0.3300)	0.0000	0.9999	0.1795 (0.3478)	0.5160	0.6198	0.0322
USA	0.0000 (0.3321)	0.0000	0.9999	0.1402 (0.3501)	0.4005	0.6992	0.0197

Table 28. Regression table: Dependent variable is the scaled node strength and the independent variable is the scaled number of employees (2008-09). *** : significant at 1%, **: at 5%, *: at 10%.

Countries	β_0	Tstat	Pvalue	β_1	Tstat	Pvalue	Rsquare
Australia	0.0000 (0.3379)	0.0000	0.9999	0.3177 (0.3584)	0.8863	0.4049	0.1009
Belgium	0.0000 (0.3806)	0.0000	0.9999	-0.0826 (0.4069)	-0.2030	0.8459	0.0068
Canada	0.0000 (0.2963)	0.0000	0.9999	0.2058 (0.3095)	0.6651	0.5210	0.0424
Denmark	0.0000 (0.3132)	0.0000	0.9999	0.3575 (0.3302)	1.0826	0.3105	0.1278
Finland	- -						
France	0.0000 (0.2516)	0.0000	0.9999	0.6613 (0.2652)	2.4937	0.0373**	0.4374
Germany	0.0000 (0.2867)	0.0000	0.9999	0.5189 (0.3022)	1.7169	0.1243	0.2693
Greece	0.0000 (0.3354)	0.0000	0.9999	-0.0161 (0.3535)	-0.0455	0.9648	0.0002
Hong Kong	0.0000 (0.2839)	0.0000	0.9999	0.5325 (0.2992)	1.7796	0.1130	0.2836
India	0.0000 (0.3086)	0.0000	0.9999	0.3916 (0.3253)	1.2038	0.2631	0.1534
Indonesia	0.0000 (0.2719)	0.0000	0.9999	0.5854 (0.2866)	2.0422	0.0754*	0.3427
Japan	0.0000 (0.2935)	0.0000	0.9999	0.4840 (0.3094)	1.5643	0.1564	0.2342
Malaysia	0.0000 (0.2819)	0.0000	0.9999	0.5418 (0.2972)	1.8232	0.1057	0.2935
Netherlands	0.0000 (0.3155)	0.0000	0.9999	0.4650 (0.3346)	1.3896	0.2073	0.2162
Norway	0.0000 (0.2463)	0.0000	0.9999	0.6789 (0.2596)	2.6153	0.0309**	0.4609
Philippines	0.0000 (0.4841)	0.0000	0.9999	0.3483 (0.5412)	0.6437	0.5656	0.1213
Portugal	0.0000 (0.3257)	0.0000	0.9999	0.5221 (0.3482)	1.4997	0.1844	0.2726
Qatar	- -						
Saudi Arabia	- -						
South Africa	0.0000 (0.3635)	0.0000	0.9999	0.3063 (0.3886)	0.7882	0.4606	0.0938
Spain	0.0000 (0.4008)	0.0000	0.9999	0.6306 (0.4481)	1.4071	0.2541	0.3976
Sri Lanka	0.0000 (0.2912)	0.0000	0.9999	0.4004 (0.3054)	1.3108	0.2224	0.1603
Sweden	0.0000 (0.3202)	0.0000	0.9999	0.4389 (0.3396)	1.2923	0.2373	0.1926
Switzerland	0.0000 (0.3072)	0.0000	0.9999	0.2565 (0.3222)	0.7960	0.4465	0.0658
Thailand	0.0000 (0.3598)	0.0000	0.9999	-0.3354 (0.3846)	-0.8722	0.4167	0.1125
UK	0.0000 (0.2450)	0.0000	0.9999	0.6830 (0.2583)	2.6445	0.0295**	0.4664
USA	0.0000 (0.2784)	0.0000	0.9999	0.5578 (0.2934)	1.9010	0.0938*	0.3112

Table 29. Correlation between average return correlations of each sector with all other sectors, and size variables of the corresponding sectors, across countries. Average correlation across sectors was computed using simple average and the log10 size factors were used for computing the overall correlation table below for 2015-16 dataset.

Countries	Mcap	Revenue	Employees
Australia	0.27	0.47	0.40
Belgium	0.49	0.88	0.87
Canada	0.08	0.17	0.17
Denmark	0.79	0.66	0.65
Finland	0.77	0.41	0.52
France	0.72	0.76	0.94
Germany	0.84	0.55	0.68
Greece	-0.58	-0.30	-0.67
Hong Kong	0.48	0.67	0.74
India	0.26	0.22	0.17
Indonesia	0.79	0.74	0.39
Japan	0.31	0.10	0.54
Malaysia	0.95	0.97	0.52
Netherlands	0.65	0.72	0.86
Norway	0.70	0.78	0.77
Philippines	0.96	0.70	0.51
Portugal	0.76	0.88	0.78
Qatar	0.64	-0.00	-
Saudi Arabia	0.45	-	-
South Africa	0.09	0.53	0.37
Spain	0.19	0.27	0.29
Sri Lanka	0.68	0.69	0.71
Sweden	0.79	0.91	0.87
Switzerland	0.86	0.82	0.75
Thailand	0.59	0.60	-
UK	0.44	0.30	0.67
USA	0.62	0.63	0.80

Table 30. Correlation between average return correlations of each sector with all other sectors, and size variables of the corresponding sectors, across countries. Average correlation across sectors was computed using simple average and the log10 size factors were used for computing the overall correlation table below for 2012-13 dataset.

Countries	Mcap	Revenue	Employees
Australia	0.44	0.54	0.57
Belgium	0.52	0.58	0.48
Canada	0.42	0.41	0.10
Denmark	0.81	0.44	0.73
Finland	0.46	0.56	0.57
France	0.62	0.75	0.59
Germany	0.62	0.34	0.43
Greece	-0.42	-0.18	-0.41
Hong Kong	0.54	0.79	0.82
India	0.00	0.14	0.03
Indonesia	0.76	0.81	0.50
Japan	0.24	0.03	0.54
Malaysia	0.93	0.90	0.93
Netherlands	0.61	0.58	0.69
Norway	0.70	0.63	0.75
Philippines	0.91	0.74	0.37
Portugal	0.82	0.33	0.43
Qatar	0.37	-0.04	-
Saudi Arabia	0.57	0.42	-
South Africa	0.54	0.74	0.68
Spain	0.88	0.63	-0.10
Sri Lanka	0.26	0.39	0.27
Sweden	0.25	0.40	0.31
Switzerland	0.67	0.68	0.70
Thailand	0.69	0.56	0.65
UK	0.41	0.43	0.79
USA	0.68	0.71	0.64

Table 31. Correlation between average return correlations of each sector with all other sectors, and size variables of the corresponding sectors, across countries. Average correlation across sectors was computed using simple average and the log10 size factors were used for computing the overall correlation table below for 2008-09 dataset.

Countries	Mcap	Revenue	Employees
Australia	0.24	0.41	0.20
Belgium	0.02	-0.08	-0.02
Canada	0.05	0.28	0.08
Denmark	0.22	0.36	0.44
France	0.51	0.78	0.66
Germany	0.66	0.52	0.39
Greece	-0.28	-0.53	-0.34
Hong Kong	0.48	0.70	0.84
India	0.71	0.51	0.50
Indonesia	0.40	0.35	0.38
Japan	0.02	0.15	0.25
Malaysia	0.94	0.92	0.33
Netherlands	0.88	0.79	0.88
Norway	0.71	0.80	0.81
Philippines	0.76	0.61	0.17
Portugal	0.79	0.74	0.63
Qatar	0.40	-0.35	-
Saudi Arabia	0.42	0.22	-
South Africa	0.36	0.63	0.58
Spain	0.24	0.55	0.50
Sri Lanka	0.46	0.37	0.04
Sweden	0.31	0.55	0.49
Switzerland	0.38	0.33	0.36
Thailand	0.08	0.03	-0.11
UK	-0.06	0.10	0.48
USA	0.19	0.20	0.51
